# Patients’ experience on pain outcomes after hip arthroplasty: insights from an information tool based on registry data

**DOI:** 10.1186/s12891-024-07357-6

**Published:** 2024-04-01

**Authors:** Gianluca Fabiano, Sophie Cole, Christophe Barea, Stéphane Cullati, Thomas Agoritsas, Nils Gutacker, Alan Silman, Didier Hannouche, Anne Lübbeke, Rafael Pinedo-Villanueva

**Affiliations:** 1 Nuffield Department of Orthopaedics, Rheumatology and Musculoskeletal Sciences, University of Oxford, Oxford, UK; 2 Division of Orthopaedics & Trauma Surgery, University Hospitals of Geneva, Geneva, Switzerland; 3 Quality of Care Service, University Hospitals of Geneva, Geneva, Switzerland; 4 Population Health Laboratory (#PopHealthLab), University of Fribourg, Fribourg, Switzerland; 5 Division General Internal Medicine, University Hospitals of Geneva, Geneva, Switzerland; 6 Department of Health Research Methods, Evidence, and Impact, McMaster University, Ontario, Canada; 7 MAGIC Evidence Ecosystem Foundation, Lovisenbergetta, 17C Oslo Norway; 8 Centre for Health Economics, University of York, York, UK; 9 Oxford NIHR Biomedical Research Centre, Oxford, UK

**Keywords:** Total hip arthroplasty, Patient reported outcome measures, Information tool, Pain management, Shared decision making

## Abstract

**Background:**

Arthroplasty registries are rarely used to inform encounters between clinician and patient. This study is part of a larger one which aimed to develop an information tool allowing both to benefit from previous patients’ experience after total hip arthroplasty (THA). This study focuses on generating the information tool specifically for pain outcomes.

**Methods:**

Data from the Geneva Arthroplasty Registry (GAR) about patients receiving a primary elective THA between 1996 and 2019 was used. Selected outcomes were identified from patient and surgeon surveys: pain walking, climbing stairs, night pain, pain interference, and pain medication. Clusters of patients with homogeneous outcomes at 1, 5, and 10 years postoperatively were generated based on selected predictors evaluated preoperatively using conditional inference trees (CITs).

**Results:**

Data from 6,836 THAs were analysed and 14 CITs generated with 17 predictors found significant (*p* < 0.05). Baseline WOMAC pain score, SF-12 self-rated health (SRH), number of comorbidities, SF-12 mental component score, and body mass index (BMI) were the most common predictors. Outcome levels varied markedly by clusters whilst predictors changed at different time points for the same outcome. For example, 79% of patients with good to excellent SRH and less than moderate preoperative night pain reported absence of night pain at 1 year after THA; in contrast, for those with fair/poor SHR this figure was 50%. Also, clusters of patients with homogeneous levels of night pain at 1 year were generated based on SRH, Charnley, WOMAC night and pain scores, whilst those at 10 years were based on BMI alone.

**Conclusions:**

The information tool generated under this study can provide prospective patients and clinicians with valuable and understandable information about the experiences of “patients like them” regarding their pain outcomes.

**Supplementary Information:**

The online version contains supplementary material available at 10.1186/s12891-024-07357-6.

## Introduction

Total hip arthroplasty (THA) is the most effective treatment to reduce pain in patients with advanced-stage hip osteoarthritis (OA) [1]. Although most patients report great improvement in pain relief, many have concerns about risks and questions about the expected benefits and harms of the operation. Patients’ expectations have been shown to predict postoperative pain [2] and vary according to patients’ demographic as well as clinical and socioeconomic characteristics [3, 4]. It is also known that expectations between surgeons and patients differ; for example, patients with the highest pain scores expect better outcomes than their surgeons [4]. As patients’ expectations expand to include general health as well as disease specific aspects, it is important that they are discussed and their likelihood is considered in a shared decision process [5].

Arthroplasty registries provide an invaluable repository of evidence which can be used to inform discussions between patients and clinicians about what to expect after surgery. However, to date, there are few tools for this clinical encounter which are specific to the relevant profile of the consulting patients and in a format that facilitates their use by both patient and clinician [6].

As part of a broader study whose methodology was recently published [7], we developed “Patients like me”, an information tool that uses data from previous participants of the Geneva Arthroplasty Registry (GAR) to identify predictors of outcomes found to be relevant by patients and clinicians: pain, activity, complications, and contralateral surgery. The tool allows prospective patients facing a THA to be matched to a cluster of patients like them in terms of preoperative predictors so that they and their clinicians can use reported outcomes by the corresponding cluster to support a meaningful discussion. This manuscript reports relevant methods and findings from the development of “Patients like me” specifically for pain outcomes and discusses its implications for clinical practice.

## Patients and methods

Data were extracted from the GAR, an institutional arthroplasty registry held by the Division of Orthopaedics at the Geneva University Hospitals (GUH) since 1996. The GAR prospectively collects information about patients’ demographics, life-style factors, surgical and environmental factors, complications, clinical, radiographic, and patient-reported outcomes since 1996 [8].

The process by which GAR data were used to develop the information tool has been reported in detail elsewhere [7]. Briefly, patients who underwent a primary elective THA between March 1996 and December 2019 were included in the study. Follow-up was undertaken until 31 December 2020. Participants who received a large head (diameter > 28 mm) metal-on-metal bearing, or a bilateral operation on the same day were excluded. The analysis was performed at hip-level hence patients could appear twice if they had both hips operated at different times.

### Outcomes and predictors

A survey was designed based on one-to-one interviews with patients, observation of preoperative education sessions, published literature, and inputs from surgeons. The survey covered specific questions with respect to the expected benefits before surgery and the perceived ones at 1, 5, or 10 years after THA including pain relief, recovery of sleep, the stop or a reduction of medications, and pain interfering in daily life including return to leisure and social activities. A sample of 379 patients was randomly selected from the GAR to be sent the survey, 72.6% of whom completed it, as well as a convenient sample of seven hip surgeons from GUH [7].

Five pain outcomes were selected: pain whilst walking, pain from going up or down stairs, pain at night, pain interfering with daily activities, and taking pain medication. Potential predictors available in the GAR for each of the five pain outcomes were selected a priori following iterative discussions with clinical experts and review of the existing literature reporting preoperative determinants of THA outcomes [9–11].

These included both demographic and clinical characteristics measured before the operation. Variables used as measures for each outcome and their predictors are detailed in the Additional file (Sect. 1).

### Statistical analysis

Conditional inference trees (CIT) analysis was used to generate classification algorithms for each of the five pain outcomes at 1, 5, and 10 years postoperatively, corresponding to the follow-up time points used by the GAR. CIT employs regression methods to identify predictors that classify the population into subgroups with similar outcome levels that are at the same time significantly different between subgroups [12]. It does so by identifying variables that are increasingly less important (as measured by association coefficients) to improving the classification until a point is reached when additional variables no longer have discriminatory power. When a significant association is found (*p* < 0.05), the corresponding cut-off value identified by the CIT algorithm splits one node of the current subpopulations into two child nodes such that outcome values are significantly different between the two child nodes. This results in a tree that grows as more splits are defined, until no other predictor leads to significantly different child nodes [13]. Findings from each CIT analysis are reported in the form of a tree showing statistically significant predictors in corresponding order with their respective p-values. Each branch terminates in a node, representing a subgroup of the population with a summary of outcome values for that cluster.

Methods for handling missing data are detailed in the Additional file (Sect. 3).

Internal validity was assessed by generating 1000 bootstrap samples of equal size to the original sample with the entire analysis re-done for each sample separately. Predictors from the main analysis were compared to the frequency of predictor identified in the 1000 bootstrapped CITs. Further details about internal validation can be found in the Additional file (Sect. 4).

The analysis was conducted using the ‘ctree’ function in R v.4.0.3 [13, 14].

## Results

A total of 6,836 operations were included in the analysis. Demographic characteristics of corresponding patients have been reported elsewhere [7]. The sample had slightly more women (56.8%) than men and mean age was 68.9 (SD = 12.2) years. Indication for surgery was mostly primary osteoarthritis (82%).

**Fig. 1:**
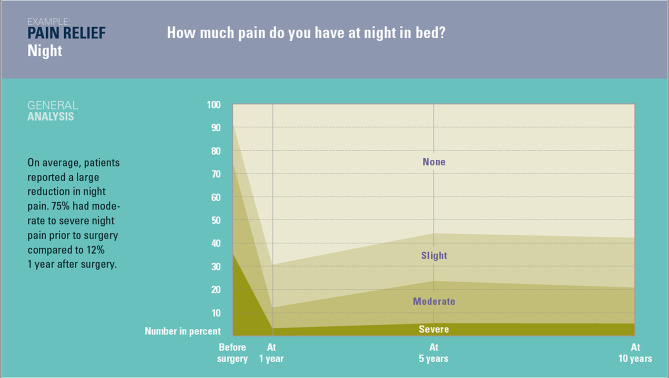
Night pain levels before and after surgery (all patients)

Reported pain levels before and after surgery for all pain outcome measures are reported in Table 1. Overall, large reductions in pain levels were observed after surgery for most patients and across the five outcomes, largely sustained over the following 10 years. Figure 1 shows the trajectory of night pain levels from before to 10 years after THA as an example of the general improvement observed in all pain outcomes for the entire cohort. Table 2 in the Additional file reports the values at each time point for night pain as shown in Fig. 1 as well as for all other outcomes.

**Table 1:** Pain outcomes at baseline and post-operatively

Pain outcomes	Number (%) *			
	Baseline (pre-op)	1-year post-op	5-years post-op	10-years post-op
**Pain during walking (WOMAC – question 1)**				
**“How bad is the pain in your hip when walking on a flat surface?”**				
None	60 (1.57)	1293 (66.34)	1339 (46.74)	622 (47.48)
Slight	314 (8.20)	354 (18.16)	607 (21.19)	269 (20.53)
Moderate	1425 (37.22)	233 (11.95)	668 (23.32)	312 (23.82)
Severe	2030 (53.02)	69 (3.54)	251 (8.76)	107 (8.17)
Ineligible **	1608	4242	2364	4530
Missing	1399	645	1607	996
**Pain using stairs (WOMAC – question 2)**				
**“How bad is the pain in your hip when you go up or down the stairs?”**				
None	62 (1.62)	1048 (53.83)	987 (34.55)	463 (35.48)
Slight	195 (5.09)	441 (22.65)	656 (22.96)	284 (21.76)
Moderate	982 (25.65)	290 (14.89)	681 (23.84)	310 (23.75)
Severe	2589 (67.63)	168 (8.63)	533 (18.66)	248 (19.00)
Ineligible **	1608	4242	2364	4530
Missing	1400	647	1615	1001
**Night pain (WOMAC – question 3)**				
**“How bad is the pain in your hip at night, in bed?”**				
None	296 (7.73)	1351 (69.32)	1601 (55.82)	757 (57.74)
Slight	655 (17.11)	361 (18.51)	590 (20.57)	282 (21.51)
Moderate	1508 (39.38)	174 (8.93)	522 (18.20)	203 (15.48)
Severe	1370 (35.78)	63 (3.23)	155 (5.40)	69 (5.26)
Ineligible **	1608	4242	2364	4530
Missing	1399	645	1604	995
**Pain interference (SF-12 – question 8)**				
**“In the past 4 weeks, and because of your emotional state (such as feeling sad, nervous, or depressed), how much did your physical pain limit you in your work or household activities?”**				
Not at all	64 (1.67)	767 (39.78)	800 (27.73)	311 (23.72)
A little bit	345 (9.00)	497 (25.78)	689 (23.88)	316 (24.10)
Moderately	1152 (30.05)	398 (20.64)	862 (29.88)	420 (32.04)
Quite a bit	1556 (40.58)	215 (11.15)	417 (14.45)	212 (16.17)
Extremely	717 (18.70)	51 (2.65)	117 (4.06)	52 (3.97)
Ineligible **	1608	4249	2373	4534
Missing	1394	659	1578	991
**Pain medication**				
**“Do you take pain medication?”**				
Yes	1338 (82.49)	467 (25.42)	530 (19.13)	259 (21.95)
Sometimes	0 (0.00)	367 (19.98)	396 (14.29)	198 (16.78)
No	284 (17.51)	1003 (54.60)	1845 (66.58)	723 (61.27)
Ineligible **	4333	4255	2373	4537
Missing	881	744	1692	1119

*Percentages are calculated based on total number of responses received as denominator, which exclude missing and ineligible

**Participants who could not complete the questionnaire because (a) it had not been introduced by the time they reached the follow-up point, (b) they did not reach the number of follow-up years at which the measure was collected, or (c) they died

### Conditional inference tree analysis

A total of 14 of the possible 15 CITs were generated; the tree for pain whilst walking at 10 years post-operatively was not produced because no variable was found to predict separate homogeneous groups for it. The resulting 14 trees are shown in Figs. 3–15 of the Additional file.

**Fig. 2:**
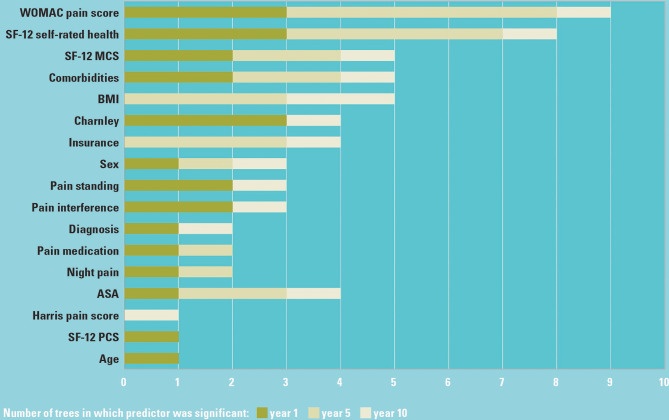
Significant (*p* < 0.05) predictors most commonly identified for all pain outcomes

A total of 17 variables out of 24 were identified as significant predictors across the five pain outcomes at 1, 5, or 10 years after THA (Fig. 2). WOMAC baseline pain score, SF-12 self-rated health (SRH), comorbidity count, SF-12 MCS, and BMI were the most common predictors determining the outcome clusters into which a patient was placed for all five pain outcomes.

#### Night pain

**Fig. 3:**
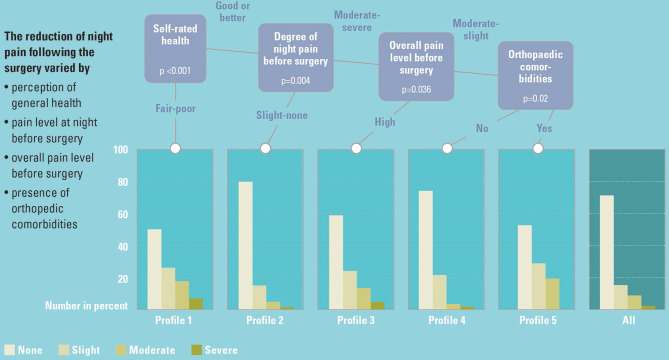
Conditional Inference Tree (CIT) for night pain at year 1

Night pain at each time point was predicted by preoperative SRH, WOMAC night pain, WOMAC pain score, BMI, and Charnley (Fig. 3). At 1 year, five nodes (clusters) were identified reporting probabilities of no night pain that varied between 79.4% in the better-off cluster to 49.8% in the worst. Lower levels of night pain were observed alongside higher baseline SRH, lower pain level (day and night) prior to surgery, and fewer orthopaedic comorbidities (Charnley A or B). At 5 years, the probability of reporting no night pain varied between 27.4% and 77.0% across the four clusters. Night pain was lower when SRH was higher and pain level lower prior to surgery. At 10 years only two clusters were identified depending only on BMI: no night pain was reported by 60.6% of those with BMI ≤ 30.7 and by 45.7% of those with BMI > 30.7.

##### Note

The cut-offs on relevant predictors were identified based on the imputed dataset, while the distribution of the outcome variable was derived using observed data.

#### Walking pain

Pain whilst walking was predicted by preoperative SF-12-MCS, SRH, pain interference, WOMAC pain score, BMI, and type of insurance (a surrogate measure of socioeconomic status). At 1-year, only two clusters were identified depending on preoperative pain interference: no pain whilst walking was reported by 74.0% of those with “moderate” or less pain interference and by 62.4% of those with “quite a bit” of or greater pain interference at baseline. At 5 years, six clusters were identified with a probability of reporting no pain whilst walking varying from 33.1 to 61.6%. Lower levels of pain whilst walking were observed alongside less pain during the day (WOMAC pain score), lower BMI score, higher SF-12-MCS and SRH, and having private insurance (i.e. higher socioeconomic status) prior to surgery. At 10 years post-surgery, no significantly different groups were generated by the CIT analysis.

#### Stairs

Pain whilst climbing up and down the stairs was predicted by preoperative WOMAC pain score, WOMAC pain standing, SF-12-MCS, SRH, pain interference, ASA, and insurance type. At 1-year post-surgery, five clusters were identified reporting probabilities of no pain whilst using the stairs that varied between 40.5% for the worse-off cluster and 71.4% for the better-off. Lower levels of pain whilst climbing up and down the stairs were observed alongside lower pain levels during the day (WOMAC pain score), higher SRH, less pain standing, and lower pain interference prior to surgery. At year 5, the number of participants reporting no pain using the stairs ranged across the seven clusters from 23.2 to 68.0% (66/97). Again, lower levels of pain whilst climbing up and down the stairs were observed alongside lower overall pain levels, higher SRH, having private insurance (higher socio-economic status), and lower ASA grade. At year 10, four clusters were identified with patients reporting no pain whilst climbing up and down the stairs ranging between 20.8% (5/24) and 60.7% (17/28). Lower levels of pain were associated with less pain during the day, pain standing and higher SRH at baseline.

#### Pain interference

At 1, 5, and 10 years after surgery, the level of pain interference was predicted by the preoperative SF-12-MCS, SF-12 pain interference, SRH score, WOMAC pain total score, WOMAC pain standing, BMI, comorbidities, Charnley, and ASA grade. At 1 year, seven clusters were identified where no pain interference was reported by 18.6% of the worst-off cluster and 60.3% of the better-off. Those who reported less pain interference also reported at baseline higher SRH, lower levels of pain standing, a higher SF-12-MCS, fewer comorbidities, a lower ASA grade, and fewer orthopaedic comorbidities (Charnley A or B). At 5 years, probabilities of reporting no pain interference ranged from 14.9 to 57.1% across the seven clusters. Those who reported lower levels of pain interference at 5 years had at baseline less pain during the day, higher SRH, lower BMI, lower ASA grade, and fewer comorbidities. At 10 years post-surgery, only two clusters were identified depending only on preoperative pain interference: one where 27.6% of patients reported no pain interference when they had less (“quite a bit” or less) pain interference before surgery, and 11.8% (11/93) who reported no pain interference when they had more (“extreme”) preoperative pain interference.

#### Pain medication

Taking pain medication after surgery was predicted by preoperative WOMAC pain score, SF-12-PCS, SF-12-MCS, Harris pain score, use of pain medication, age, sex, BMI, underlying diagnosis, comorbidities, Charnley, ASA grade, and type of insurance at years 1, 5, and 10. At 1 year, 15 clusters were identified and between 2.2% (1/46) and 51.0% reported use of pain medication across the clusters. For those who reported a lower probability of pain medication use they also reported less preoperative daily pain, fewer comorbidities, a higher SF-12-MCS and SF-12-PCS, no preoperative pain medication use, they were younger in age, had fewer orthopaedic comorbidities (Charnley A or B), and fewer number of underlying diagnoses. Being male was also associated with lower reported pain medication use. At year 5, use of pain medication ranged from 6.8% (8/117) to 53.3% (8/15) across the 12 clusters. Those who reported less pain medication use were more likely to be men, to have private insurance (i.e. better socioeconomic status), and also reported lower daily pain at baseline, fewer comorbidities, lower BMI, and a lower chance of using pain medication before surgery. At 10 years post-surgery, 16 clusters were identified where reported pain medication use ranged from 9.8% (8/82) to 60.0% (6/10). Lower levels of pain medication use were observed alongside the same predictors as year 1 and 5 except for SF-12-PCS and the addition of lower levels of pain (Harris score).

### Internal validation

All predictors in 10 of the 14 CITs of the main analysis also appeared in > 50% of the 1000 bootstrapped trees generated for validation. Only one of all predictors identified in the main analysis of each of the remaining four CITs was found in < 50% of the bootstrapped trees. Detailed results are reported in the Additional file (Sect. 4).

### Personalised reference points for pain outcomes

**Fig. 4:**
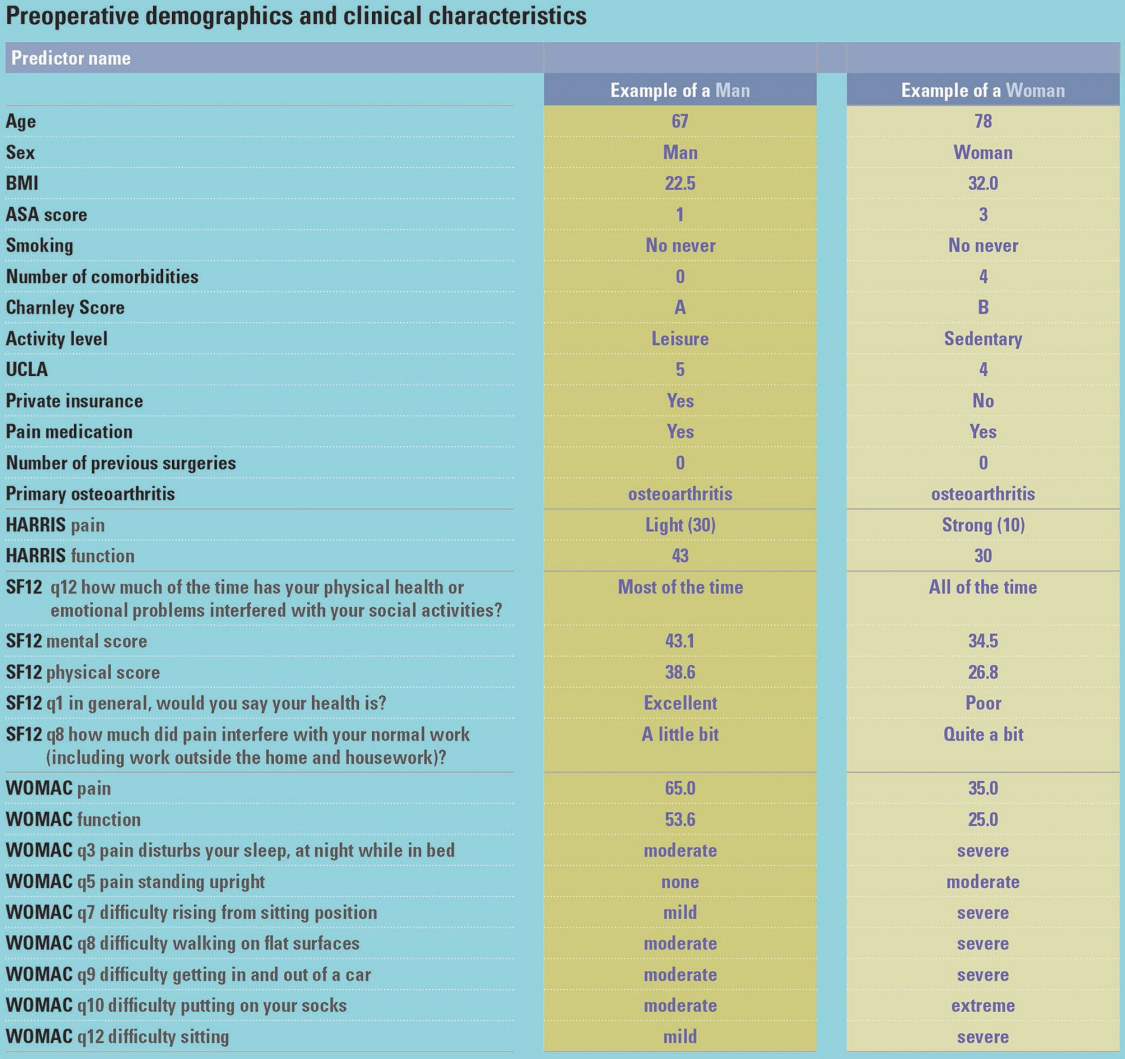
Preoperative demographic and clinical characteristics for exemplar man and woman patient

**Fig. 5:**
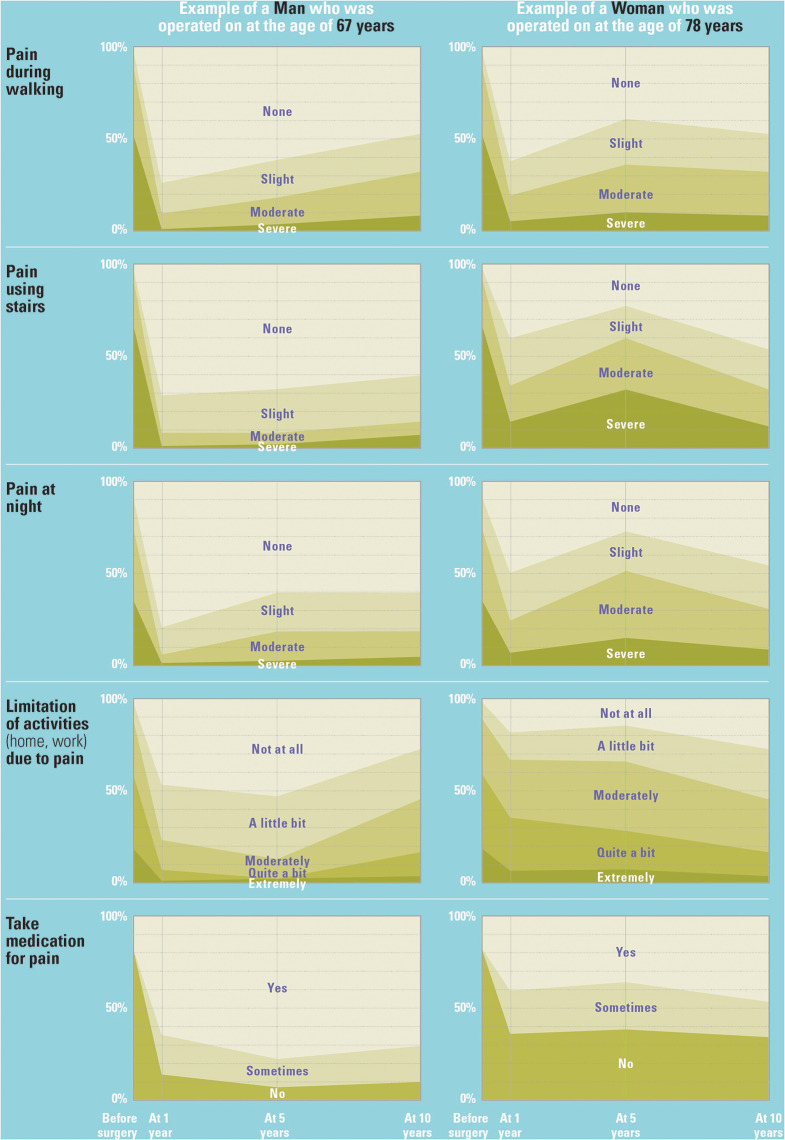
Personalised reference points of pain trajectories for exemplar man and woman patient

The generation of the above CITs allows for any patient completing the corresponding questions for all relevant pre-operative predictors to be matched to a single cluster for each of the five pain outcomes at each of the three time points. Figure 4 shows the baseline characteristics of an exemplar man and woman patient with their corresponding clusters shown in Fig. 5.

## Discussion

This work is part of a wider project aimed at developing a comprehensive tool to inform patients and clinicians based on the experience of previous patients over up to 20 years after surgery [7]. In this study we showed that most patients undergoing an elective THA report improved pain outcomes after surgery, but also that distinct clusters of varying pain outcome levels can be identified based on pre-operative predictors.

Our approach is novel in that it uses clustering methods (via CITs) instead of clinical prediction or prognostic models to generate information about what patients experience after surgery. This allowed to report cluster-associated outcomes as proportions, which is easier to interpret by patients and allows them to see the 10-year outcome trajectories of previous patients who were similar to them.

The need for greater awareness and reporting of long-term outcomes following joint replacement has been previously highlighted [18]. Few studies report proportions in addition to or instead of mean pain scores and those that do indicate that the proportion of people with an unfavourable long-term pain outcome (up to 4 years) ranges from 7 to 23% after hip replacement [15]. Our findings fall within this range. Pain at night is less frequently studied, although it is known to be a major complaint of patients prior to surgery. One study found 25% of patients undergoing THA reported sleep disturbance due to pain six months after surgery [16]. We observed a large reduction in moderate to severe night pain occurrence from 74% prior to surgery to 12% one year after surgery.

We identified a series of factors that predict patient clusters in relation to pain after surgery, many of which have been recognised before. Galea et al. [17] found that obesity, pain in other joints, anxiety/depression, and difficulty in self-care explained baseline differences in SF-36, EQ-5D, and Harris Hip score at one year. Although we used different outcomes and many more predictors, we also found obesity, lower mental health and/or health status, and higher preoperative pain levels due to other orthopaedic comorbidities to be predictors of different pain dimensions after THA.

The most frequently identified significant predictors for the 1- and 5-year pain outcomes were preoperative level of pain, a well-known essential predictor [9], and SRH. Although the latter is an important predictor of patient-reported outcomes after hip and knee arthroplasty [18], SRH remains absent or at best infrequently used in prognostic studies, possibly related to the fact that the EQ-5D is more frequently employed to measure general health in arthroplasty patients than the SF-12 [19]. Our analyses highlight the importance of SRH as an independent predictor of pain after THA. Other clusters were determined by preoperative mental health status, comorbidity count, ASA grade, BMI, Charnley disability grade, and socioeconomic status (measured via insurance), all of them previously described predictors of pain after THA [9]. Pain may decrease at varying rates over time. Subgroups with characteristics associated to a slower or a diminished recovery, such as high BMI (Fig. 2), remain at risk over extended follow-up periods. Pain may also decrease initially whilst increasing again with time. The subsequent increase can be attributed to aging and the emergence of additional comorbidities, either medical or musculoskeletal, affecting pain related to lower limb movements, pain during rest or the requirement for medication.

This study has limitations. First, as highlighted in other publications using similar methods [20], the conditional tree approach does not allow for alternative ways of splitting the predictor variables that lead to the creation of clusters, thus making the latter highly sensitive to changes in the former and the overall result potentially restricted in its replicability. The internal validity by bootstrap assessed the impact of this limitation and found resulting trees largely consistent. A second limitation is the high levels of missing data. This is a common problem of long-term cohort data which often have increasing loss to follow-up over the analysed time frame especially considering the patients’ advanced age at the time of surgery. For example, Galea et al. reported 25% of missing data over a 7-year follow-up period [17], which is similar to our study. While recognising the importance of back pain, especially in the night, this aspect was not included in our analysis. Also, patients were asked about pain medication use in general, not specifically for pain in their hip.

Prognostic models are usually assessed on their performance, such as calibration and discrimination [21]. However, the tool we developed is an information tool, not a prognostic one. A prognostic model is a mathematical equation that connects multiple predictors for an individual to the probability or risk of a particular outcome [22]. This allows their validation by comparing observed versus predicted values. The tool we developed cannot be assessed in that way as we applied clustering techniques to a group of patients who had an arthroplasty in the past into subgroups to which new patients could be matched to as a reference. Caution is therefore warranted for clinicians using this tool to highlight that it is not prognostic but informative. This is a substantial methodological difference from predictive tools such as the one by Franklin et al. [6] where patient reported outcomes and clinical risk variables are collected prior to the visit and compared against national registry data to generate individualised estimates of likely postoperative outcomes. Differently, the clusters arising from our analysis can inform the discussions between clinician and patient and make them more meaningful and relevant, for the patient especially, by conveying what the experience of patients like them has been. It is not suggesting how they might do after their surgery, but rather how others like them have, with variability that can be graphically shown in ways that are easily understood. Nevertheless, as the tool is ultimately based on regression models, their performance must be considered and assessed. As it has now been incorporated into clinical practice at the GUH and both profiles matching and actual outcomes are being collected, those data will be used in the future to assess the extent to which patient outcomes match those of their assigned patient profile (calibration) as well as how accurately the tool matches profiles indicating a higher probability of having certain levels of a pain outcome to patients who effectively experienced those (discrimination). All of this is now possible as patients at the GUH can see and discuss with their clinicians personalised reference points of pain trajectories such as those shown here for an exemplar man and woman.

The tool implementation at the GUH encompasses a patient information leaflet, a digital visualization tool for surgeons, and an infographic brochure [7]. Providing these resources has been the only prerequisite to implement the tool for any new prospective THA patient at the GUH who participates in the registry, as by doing so all necessary preoperative variables get collected and corresponding matches to previous patients like them and their outcomes are generated. Trees for all pain outcomes at the three time points are openly available via this manuscript; however, they should only be used in the context of clinical consultations leading to a potential surgery at the GUH until it is externally validated.

Plans are underway for further external validation of this work using registry data from other settings and countries to evaluate the applicability of the tool beyond the GAR. The methods used were chosen based on the large number of variables and the long-term follow-up available in this specific registry, which can make it challenging to fully replicate. However, findings from this analysis can also inform research using the same methods using data from other registries where not all but at least selected predictors of interest might be available so that their impact on pain outcomes is examined and made available to patients and clinicians.

In conclusion, by employing the appropriate methods registries can be useful sources of data to identify groups of past patients whose trajectories and experience can provide valuable information for patients “like them” who are about to undergo a THA. This information can serve as a guide for prospective patients and clinicians to have meaningful discussions about the intervention, expectations, and their future care.

### Electronic supplementary material

Below is the link to the electronic supplementary material.

Supplementary Material 1

## Data Availability

The datasets generated and/or analysed during the current study are not publicly available due to local data protection rules, but de-identified data are available from the corresponding author on reasonable request and after permission from the local ethics committee.
