# Supplementary material for: Patients’ experience on pain outcomes after hip arthroplasty: insights from an information tool based on registry data
Source: BMC Musculoskelet Disord. 2024 Apr 1;25:255. doi: 10.1186/s12891-024-07357-6 (PMC10986127; doi:10.1186/s12891-024-07357-6)
Supplement: Supplementary file 1 — Supplementary Material 1 [file 12891_2024_7357_MOESM1_ESM.docx]

**Outcomes and predictors:**

The five pain outcomes were pain whilst walking, pain from going up or down stairs, pain at night, pain interfering with daily activities, and the need to take pain medication. The Western Ontario and McMaster Universities Arthritis Index (WOMAC) pain questions was employed as a measure of pain whilst walking, from going up or down stairs, and whilst in bed at night. Due to the limited differentiation between responses of “extremely severe” and “severe” in the WOMAC questionnaire, these responses were merged into one group called “severe”. The SF-12 question about how much pain interferes with normal work was used as a measure of pain interference. Pain medication was covered by a direct question in the GAR about whether patients were taking any pain relief medication for any reason.

Predictors of the five pain outcomes were: age, sex, body mass index (BMI), comorbidity count and previous hip surgeries, underlying diagnosis, symptom duration (<1 year, 1-2 years, 2-5 years, >5 years)), ASA grade (healthy, mild systemic disease, server systemic disease, severe systemic and threat to life), Charnley disability grade (one hip, both hips, multiple joints or other disabilities; abbreviated as Charnley in the following), smoking status (never, former, current), and whether participants had public or private health insurance (as a proxy for socioeconomic status [1]) were included as potential predictors. Age and BMI were treated as continuous variables. Other eligible predictors included pain-specific variables measured at baseline such as preoperative pain visual analogue score (VAS), the WOMAC questionnaire (questions 1, 2, 3, 4 and 5 and the overall WOMAC pain score, 0-100 (=no pain)), Harris pain score (0-44 (=no pain)), and pain medication. Question 1 of the SF-12 questionnaire about self-rated health (SRH; poor, fair, good, very good, excellent), question 8 about pain interference, and the composite physical (PCS) and mental component scores (MCS) were also included. Comorbidity count, SF-12 physical and mental component scores, WOMAC pain and function and Harris pain were treated as continuous variables. The choice of potential predictors was based on published evidence reporting preoperative determinants of THA outcomes [2-4]) and expert clinical input.

Table 1: Outcomes and predictors

| **Outcomes** | **Description** |
| --- | --- |
| Pain during walking | WOMAC – question 1: “How bad is the pain in your hip when walking on a flat surface?” |
| Pain using stairs | WOMAC – question 2: “How bad is the pain in your hip when you go up or down the stairs?” |
| Night pain | WOMAC – question 3: “How bad is the pain in your hip at night, in bed?” |
| Pain interference | SF12 – question 8: “In the past 4 weeks, and because of your emotional state (such as feeling sad, nervous, or depressed), how much did your physical pain limit you in your work or household activities?” |
| Pain medication | Question during visits (year 1) and throughout questionnaire (years 5, 10) |
| **Predictors (baseline values)** | **Description** |
| Age | Age of participants on the day of the surgery |
| Sex | Female/Male |
| BMI | Body mass index - Quetelet formula (kg/m2) – treated as continuous variable [6, 7] |
| ASA grade | ASA Physical Status Classification System (healthy, mild systemic disease, server systemic disease, severe systemic and threat to life) |
| No. of comorbidities | Number of recorded comorbidities |
| Charnley | Charnley classification (A: patients have single joint arthropathy and no significant medical comorbidity, B: patients have one other joint in need of an arthroplasty, or an unsuccessful or failing arthroplasty in another joint, C: patients have multiple joints in need of arthroplasty, multiple failing arthroplasties or significant medical or psychological impairment) |
| Diagnosis | Number of diagnoses (underlying condition) |
| Smoking | Whether patients smoked (never, former, current) |
| Insurance | Whether patients had private insurance at time of surgery |
| No. of previous surgeries | Number of previous surgeries recorded in GHAR |
| OA symptom duration | Duration of symptoms in years (<1 year, 1-2 years, 2-5 years, >5 years) |
| Self-rated health | SF-12 – question 1: “Overall, do you think your health is?” (Poor, fair, good, very good, excellent) |
| Pain interference | SF-12 – question 8: “In the past 4 weeks, and because of your emotional state (such as feeling sad, nervous, or depressed), how much did your physical pain limit you in your work or household activities?” |
| Physical Composite Scale | SF-12 - Physical Composite Scale – PCS |
| Mental Health Composite Scale | SF-12 - Mental Health Composite Scale - MCS |
| VAS pain | Visual Analogue Scale |
| Pain walking | WOMAC – question 1: “How bad is the pain in your hip when walking on a flat surface?” |
| Pain stairs | WOMAC – question 2: “How bad is the pain in your hip when you go up or down the stairs?” |
| Night pain | WOMAC – question 3: “How bad is the pain in your hip at night, in bed?” |
| Pain getting up | WOMAC – question 4: “How bad is the pain in your hip when you get up from a chair or when you sit down?” |
| Pain while standing | WOMAC – question 5: ““How bad is the pain in your hip when you stand?” |
| WOMAC score | WOMAC overall score, 0-100 (=no pain) |
| Harris hip pain | Harris hip pain sub score, 0-44 (=no pain) |
| Pain medication | Question during pre-operative visit |

Table 2: Progression of pain outcomes and pre- and post-operative time points

| **Night Pain** | **Pre-op** | **%** | **Year 1** | **%** | **Year 5** | **%** | **Year 10** | **%** |
| --- | --- | --- | --- | --- | --- | --- | --- | --- |
| None | 296 | 7.73 | 1351 | 69.32 | 1601 | 55.82 | 757 | 57.74 |
| Slight | 655 | 17.11 | 361 | 18.52 | 590 | 20.57 | 282 | 21.51 |
| Moderate | 1508 | 39.38 | 174 | 8.93 | 522 | 18.2 | 203 | 15.48 |
| Severe | 1370 | 35.78 | 63 | 3.23 | 155 | 5.4 | 69 | 5.26 |
| **Stairs** | **Pre-op** | **%** | **Year 1** | **%** | **Year 5** | **%** | **Year 10** | **%** |
| None | 62 | 1.62 | 1048 | 53.83 | 987 | 34.55 | 463 | 35.48 |
| Slight | 195 | 5.09 | 441 | 22.65 | 656 | 22.96 | 284 | 21.76 |
| Moderate | 982 | 25.65 | 290 | 14.89 | 681 | 23.84 | 310 | 23.75 |
| Severe | 2589 | 67.63 | 168 | 8.63 | 533 | 18.66 | 248 | 19 |
| **Walking pain** | **Pre-op** | **%** | **Year 1** | **%** | **Year 5** | **%** | **Year 10** | **%** |
| None | 60 | 1.57 | 1293 | 66.34 | 1339 | 46.74 | 622 | 47.48 |
| Slight | 314 | 8.2 | 354 | 18.16 | 607 | 21.19 | 269 | 20.53 |
| Moderate | 1425 | 37.22 | 233 | 11.95 | 668 | 23.32 | 312 | 23.82 |
| Severe | 2030 | 53.02 | 69 | 3.54 | 251 | 8.76 | 107 | 8.17 |
| **Pain medication** | **Pre-op** | **%** | **Year 1** | **%** | **Year 5** | **%** | **Year 10** | **%** |
| No | 284 | 17.48 | 1003 | 54.6 | 1845 | 66.58 | 723 | 61.27 |
| Sometimes | NA | NA | 367 | 19.98 | 396 | 14.29 | 198 | 16.78 |
| Yes | 1341 | 82.52 | 467 | 25.42 | 530 | 19.13 | 259 | 21.95 |
| **Pain interference** | **Pre-op** | **%** | **Year 1** | **%** | **Year 5** | **%** | **Year 10** | **%** |
| Not at all | 64 | 1.67 | 767 | 39.78 | 800 | 27.73 | 311 | 23.72 |
| A little bit | 345 | 9 | 497 | 25.78 | 689 | 23.88 | 316 | 24.1 |
| Moderately | 1152 | 30.05 | 398 | 20.64 | 862 | 29.88 | 420 | 32.04 |
| Quite a bit | 1556 | 40.58 | 215 | 11.15 | 417 | 14.45 | 212 | 16.17 |
| Extremely | 717 | 18.7 | 51 | 2.65 | 117 | 4.06 | 52 | 3.97 |

1. **Missing data**

Some patients reported missing data on baseline predictors, and loss to follow-up across the ten years resulted in further increasing missing data on outcomes over time. Reasons for not completing follow-up questionnaires included death, moving away from Switzerland, refusal to participate, and poor general health. To mitigate its impact on results, imputation methods were used to predict values for missing data on BMI, comorbidity count, Charnley score, smoking status, number of previous hip surgeries, OA symptoms duration, pain VAS, pain medication, and questionnaire items (SF-12, Harris Pain Score, WOMAC). There was no need to impute values for ASA grade, diagnosis, or private insurance as these had no missing data. First, patterns of missing data were evaluated by running logistic regressions to identify whether there were observed variables associated with data being missing (missing at random). As missingness was accounted for by variables for which there was complete information (such as age, sex and ASA grade), data were considered to be largely missing at random and were hence imputed using multiple imputation by chained equations methods [5]. Chained equations used the variables with missing data as response and those identified in the logistic regressions above as well as variables considered potentially associated to them as predictors. For example, a pain outcome would be considered a reasonable predictor for another pain outcome. Observed and imputed pain medication were compared stratified by sex, age, and BMI to check that there were not clear differences (see supplementary material, Tables 2-25).

Because the CIT R package could not manage multiple imputed datasets, the first of the 50 imputed iterations was used to generate CITs after confirming that the distribution of questionnaire responses of all repeated iterations was consistent with those of the observed sample. However, the distribution of each outcome variable was ultimately derived using observed data and applying the cut-offs on relevant predictors identified in the respective CIT nodes. This allowed for groups to be defined with mitigated bias from the missing data and for the characterisation of each group to be strictly representative of the patients classified into each and their observed outcomes.

1. **Internal validation**
   1. Methods:

Internal validity was assessed by generating 1000 bootstrap samples of equal size to the original sample with the entire analysis re-done for each sample separately. Predictors from the main analysis were compared to the frequency of predictor identified in the 1000 bootstrapped CITs. Predictors that were included in at least 50% of bootstrapped CITs were considered “likely” and those included less frequently were deemed “unlikely” predictors. Further, the number of terminal nodes in the trees of the main analysis was compared to the average number of terminal nodes across the bootstrapped trees. This was done for each pain outcomes at each time point (years 1, 5 and 10).

In figure 1 we compared the predictors which appeared in our original analysis to number of the times each predictor appeared in the 1000 bootstrapped trees for each pain outcome. In Figure 2, we compare the mean size of the trees generated using the bootstrap samples to the size of the tree in the model. The bars in the plot indicate the min-max 95% percentiles.

- 1. Results:

All predictors in 10 of the 14 CITs of the main analysis also appeared in >50% of the 1000 bootstrapped trees generated for validation. Only one of all predictors identified in the main analysis of each of the remaining four CITs was found in <50% of the bootstrapped trees: pain interference for the CIT pain whilst climbing up and down the stairs at year 1, SRH for pain whilst climbing up and down the stairs at years 10, WOMAC pain score for night pain at year 1, and ASA for pain interference at year 1. Other likely variables (>50% frequency) were found in the bootstrapped sample and not identified in the main analysis; however, these were less frequent than the predictors found in the main analysis. The 15^th^ CIT of the main analysis was the one corresponding to pain whilst walking at year 10; in the validation, this outcome reported only ‘unlikely’ predictors, with the most common appearing only in 16% of the bootstrapped trees.

Nine of the CITs in the main analysis had fewer clusters (terminal nodes) than the average size of the 1000 bootstrapped trees. The largest tree size difference between the main analysis and the bootstrap validation was found in pain medication at year 1, which resulted in trees averaging 30.9 terminal nodes in the bootstrap validation compared with 15 nodes in the main analysis; night pain at year 5 with a mean of 8.6 terminal nodes in the bootstrap compared to four in the main analysis; and pain interference at year 5 with a mean of 12.5 terminal nodes in the validation compared to seven in the main analysis. A summary of predictors and a comparison of the size of trees after validation is provided Figure 2.

In some cases, we found that “likely” variables in the bootstrapped samples were not included in the model. This may be explained by the fact that on average the number of terminal nodes in the bootstrap was larger than in the analysis, which makes it more likely for bootstrapped trees to include more predictors. Furthermore, in most of the cases, the likely predictors not included in the model were less frequent than those that were included. Although the mean size of bootstrapped trees was larger than those generated in the main analysis for nine CITs, the fact that this was found together with an alignment of predictors in the validation suggests that a broader pool of data are likely to find these same predictors leading to a larger number of and yet more specific sets of profiles. We would expect that with more data collected over the years this would be mirrored in the main analysis.


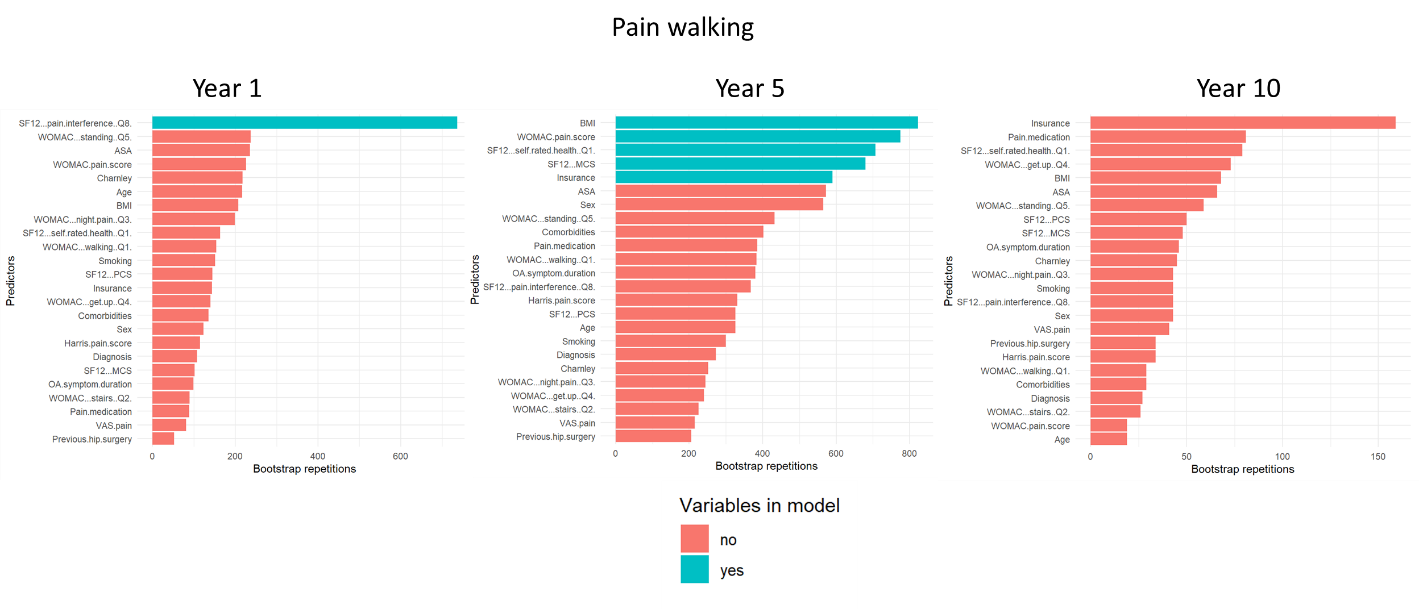


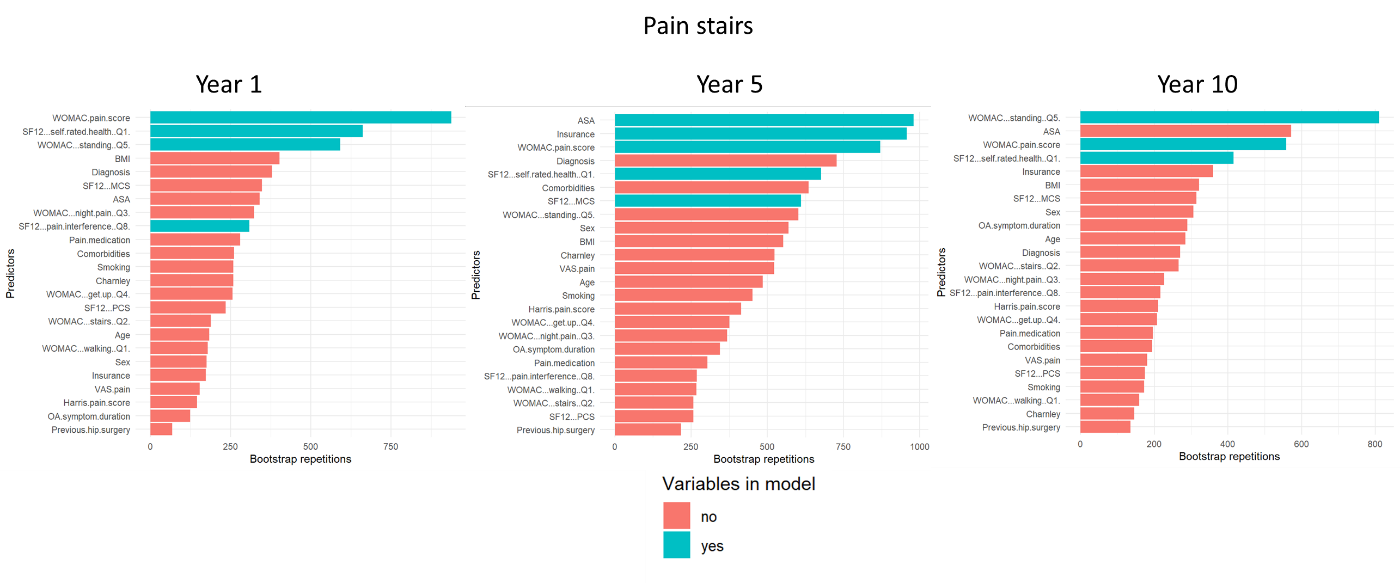


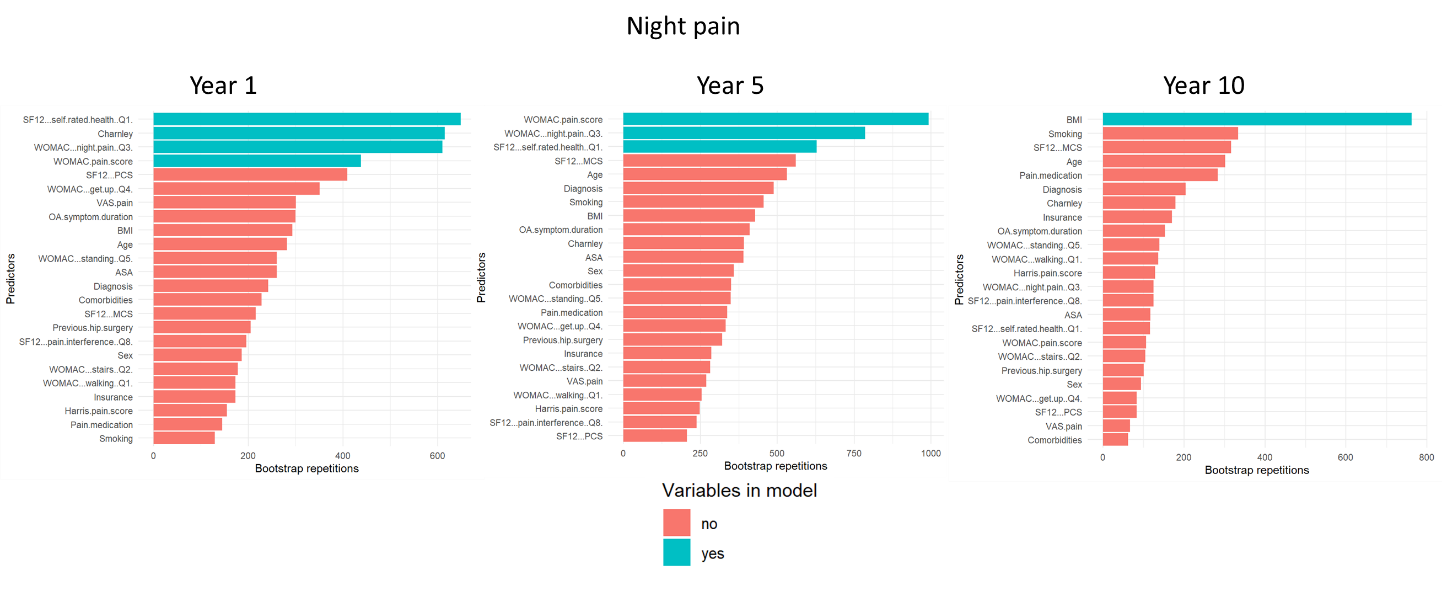


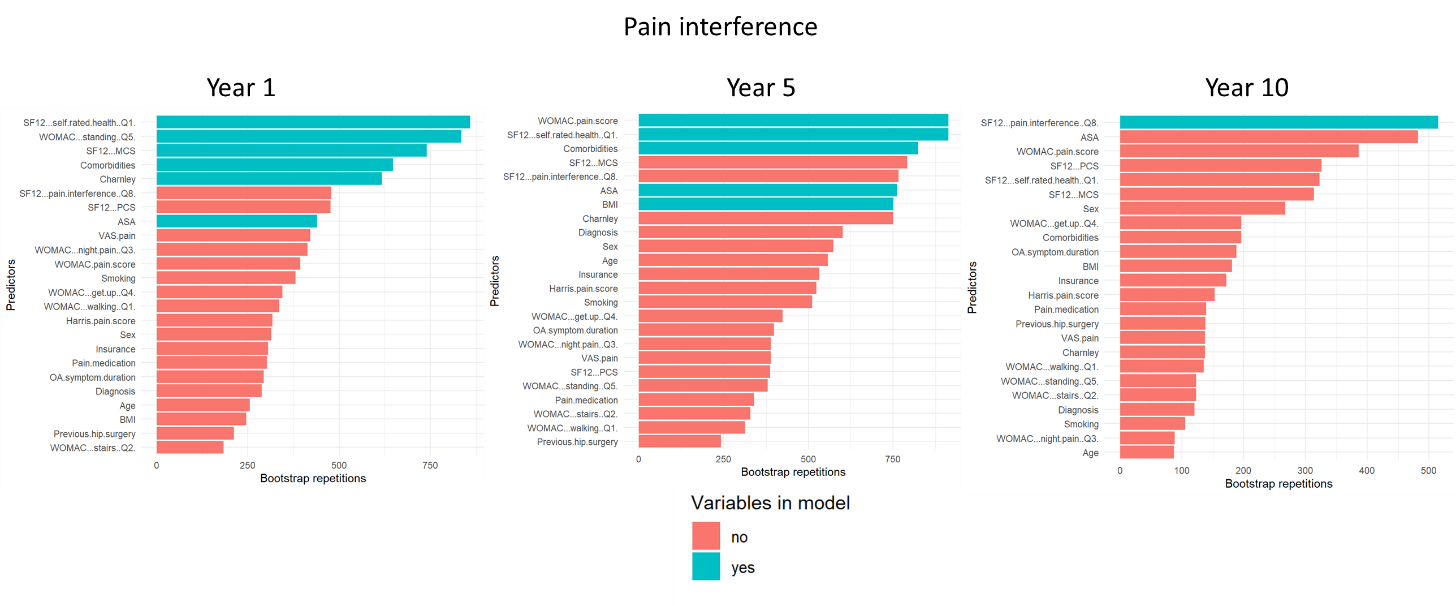

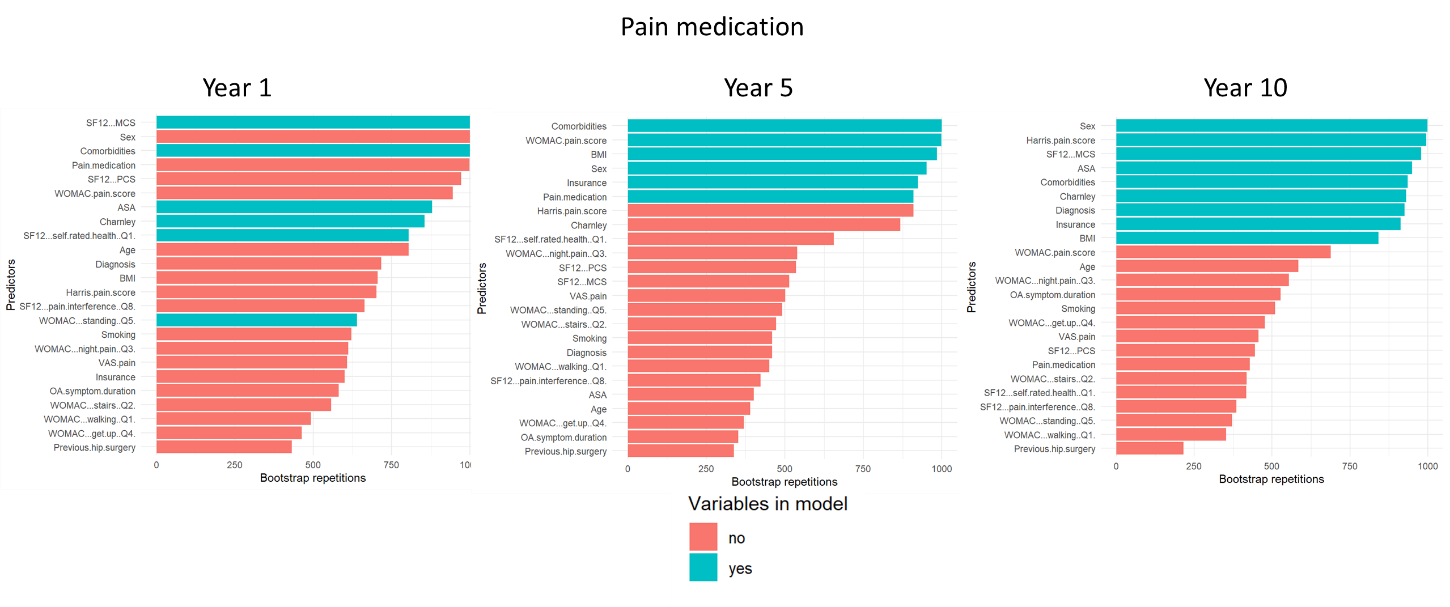


Figure 1: Model Validation, variable frequency


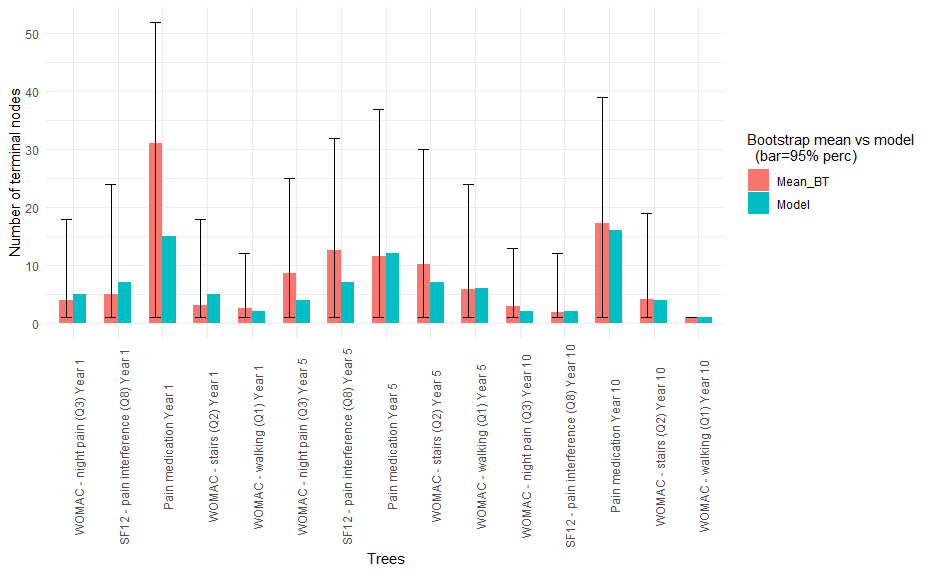


Figure 2: Model validation, tree size

1. **Results of Conditional Tree analysis**


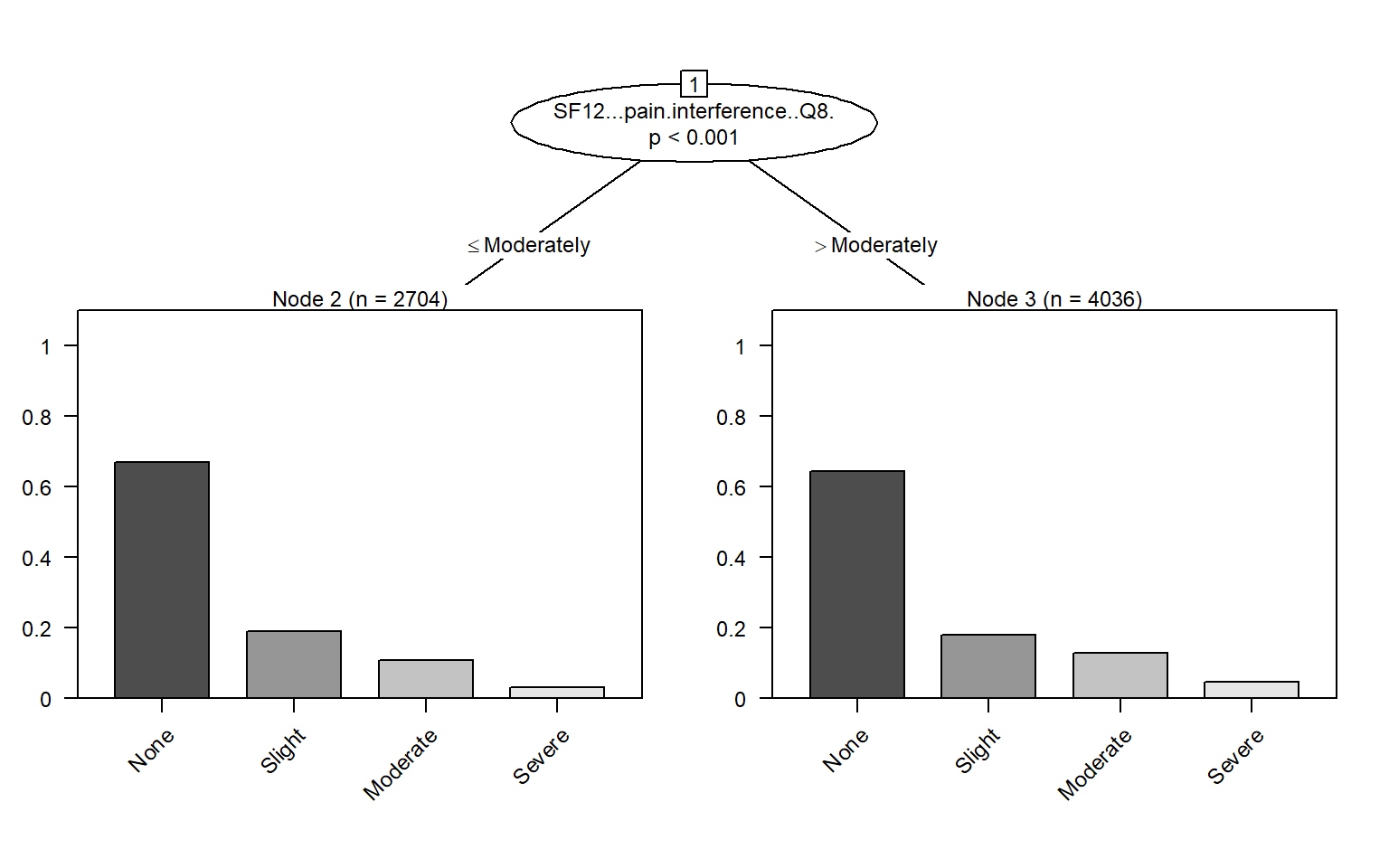


Figure 3: Pain walking - year 1


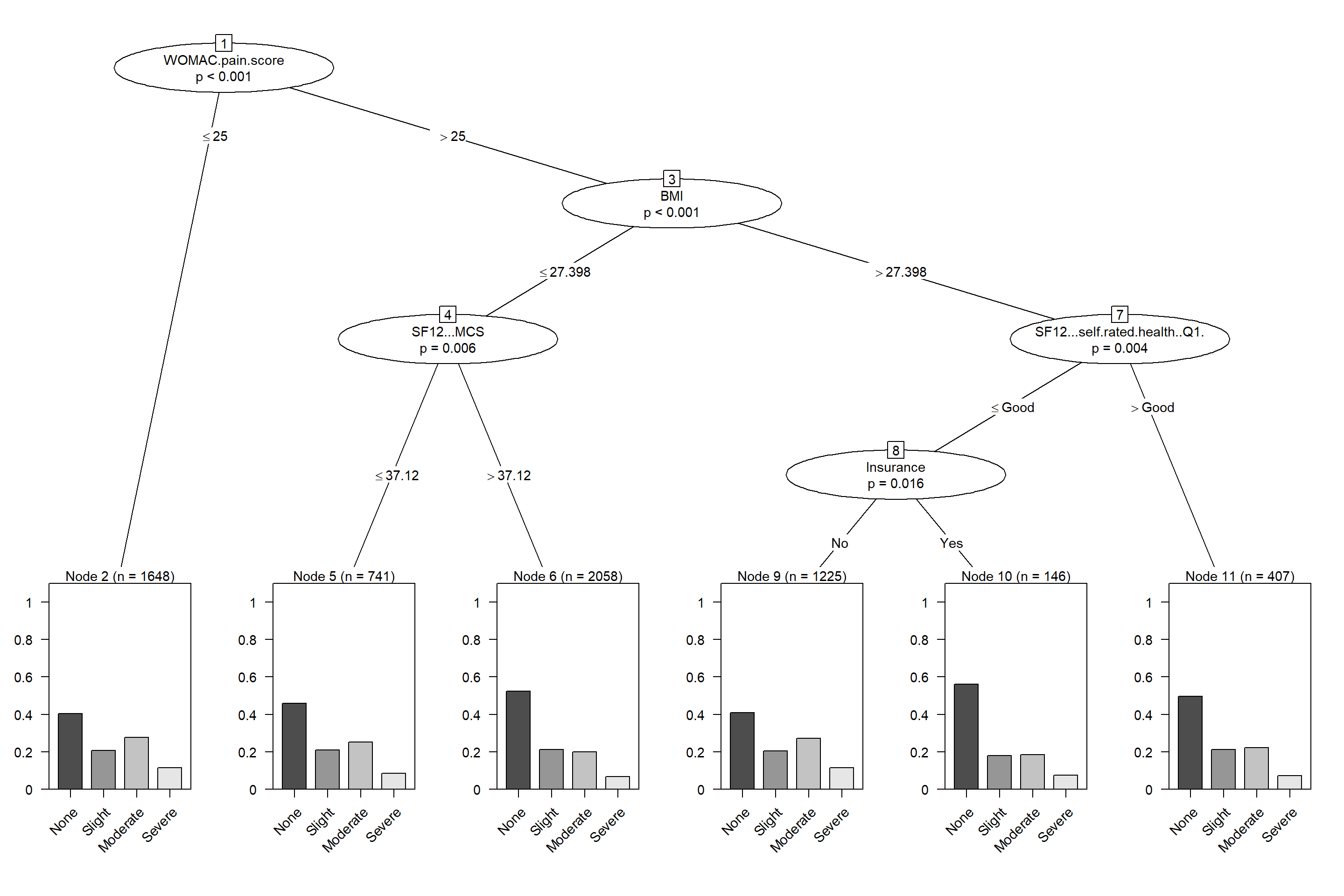


Figure 4: Pain walking – year 5


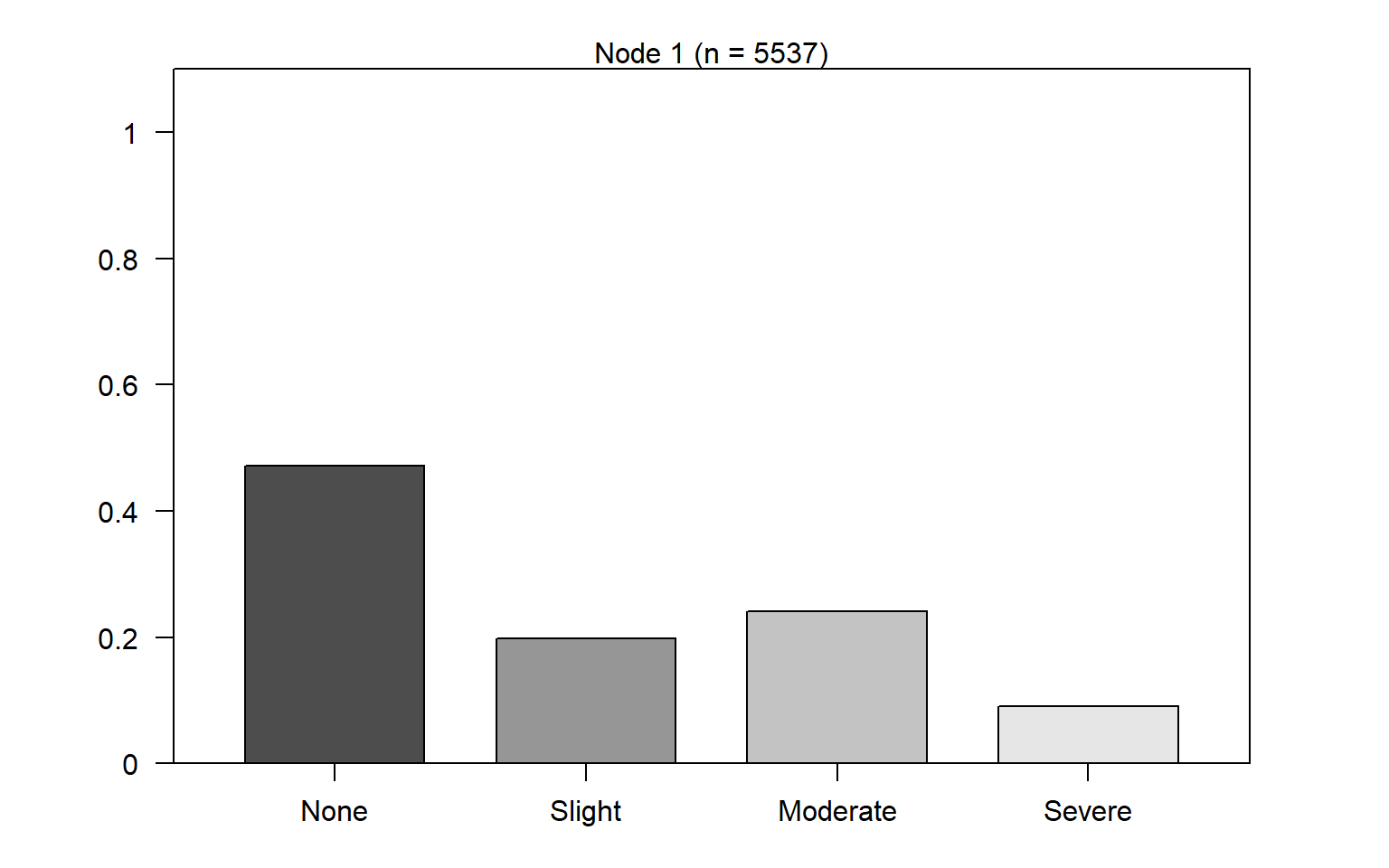


Figure 5: Pain walking – year 10


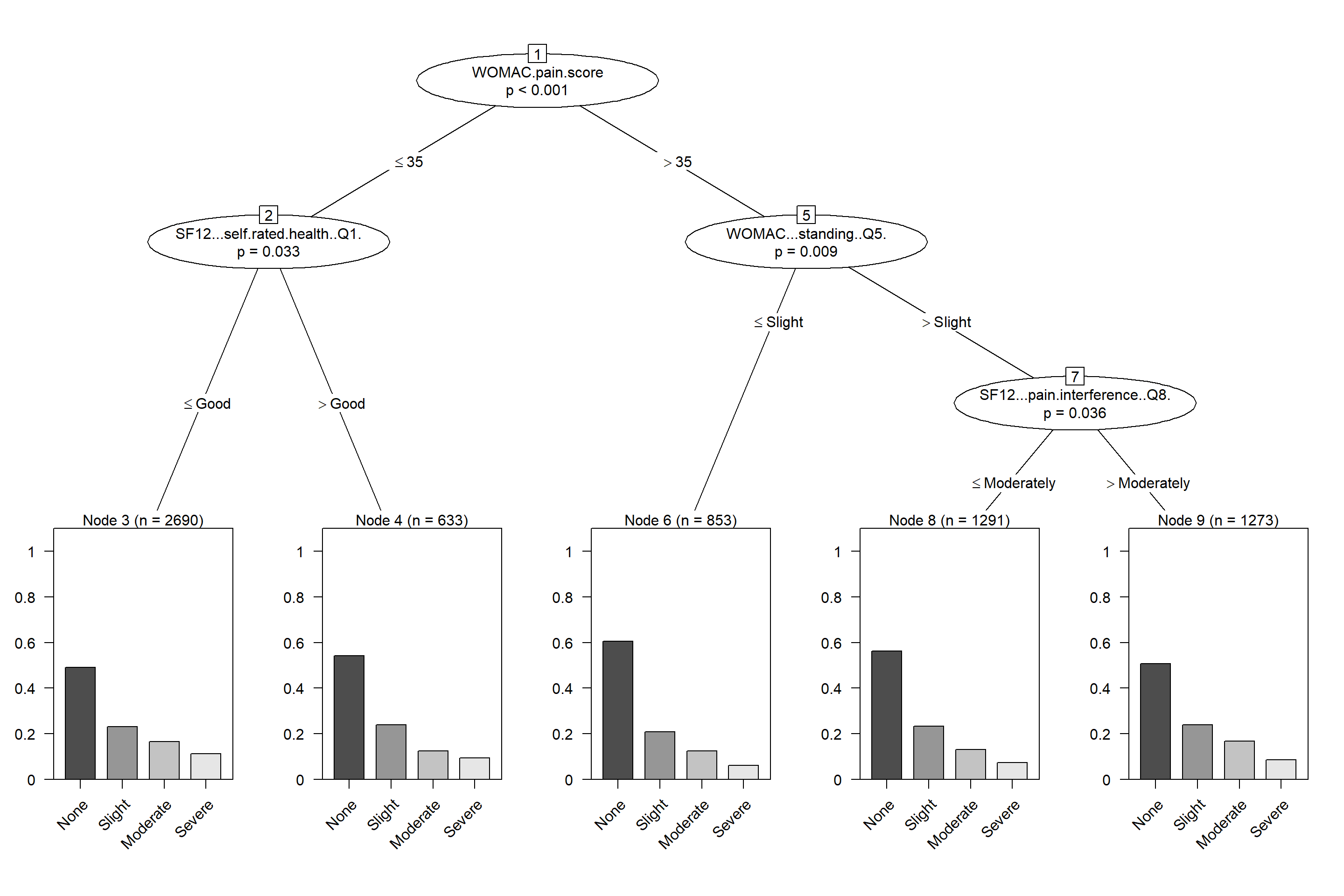


Figure 6: Stairs – year 1


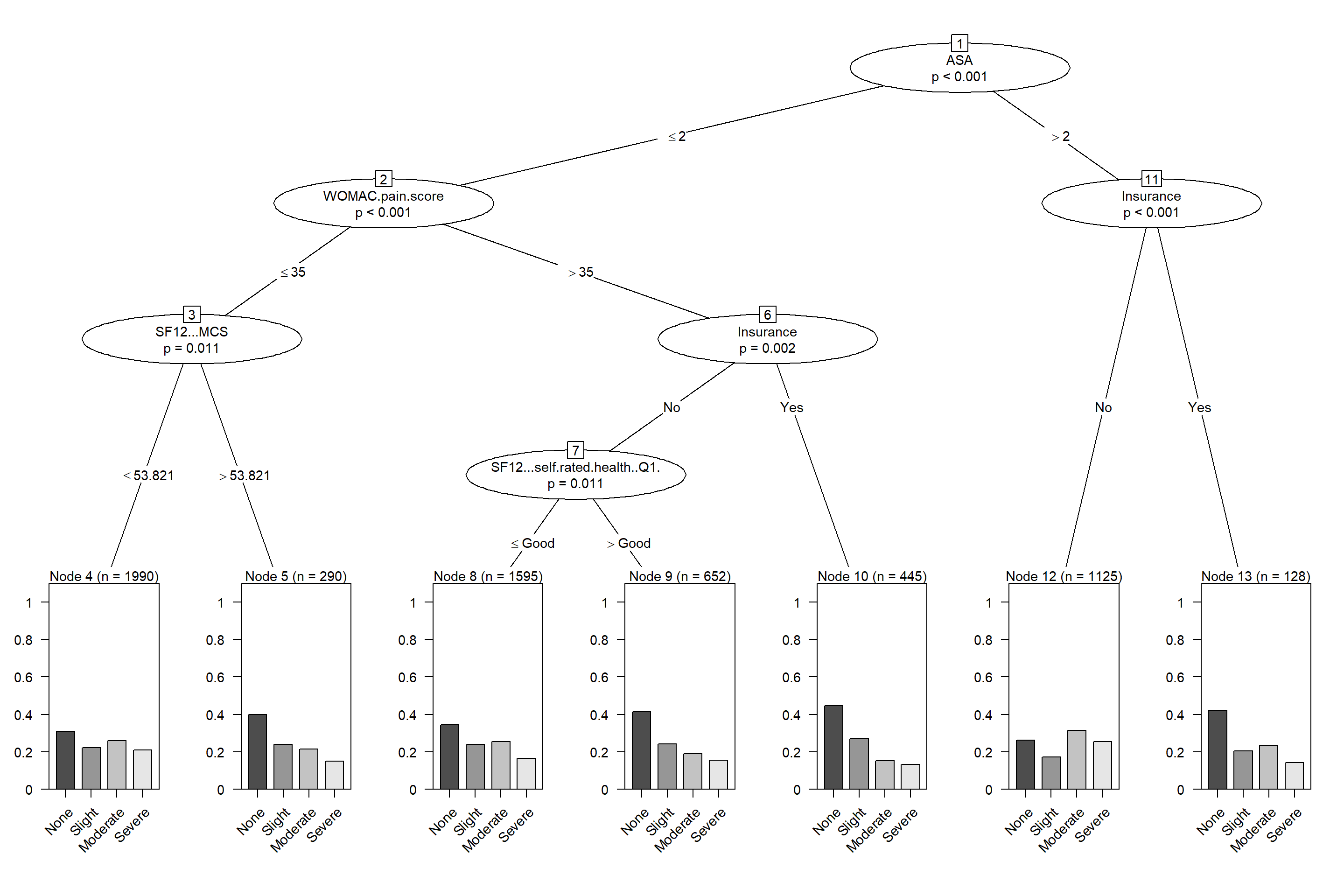


Figure 7: Stairs – year 5


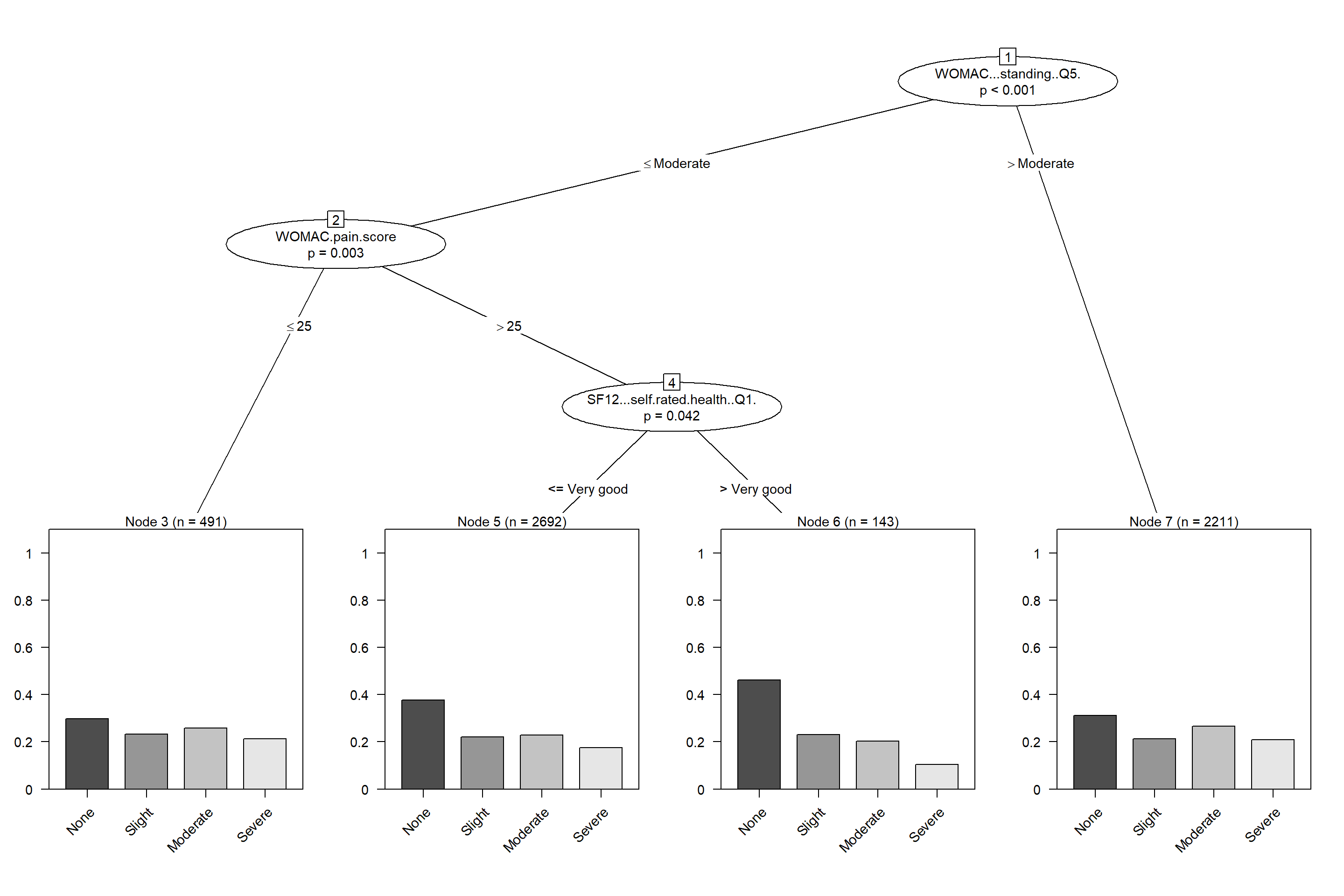


Figure 8: Stairs – year 10


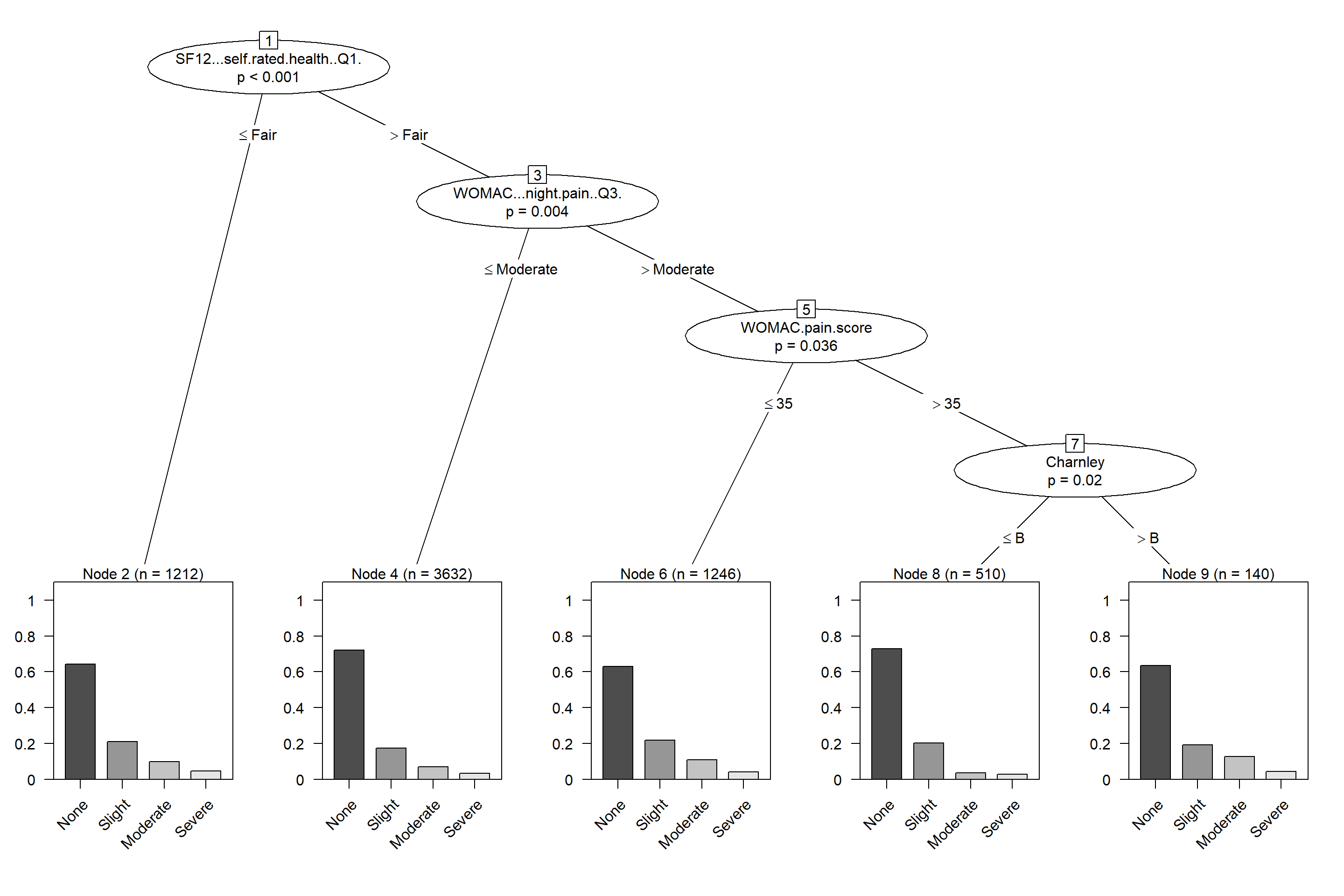


Figure 9: Night pain – year 1


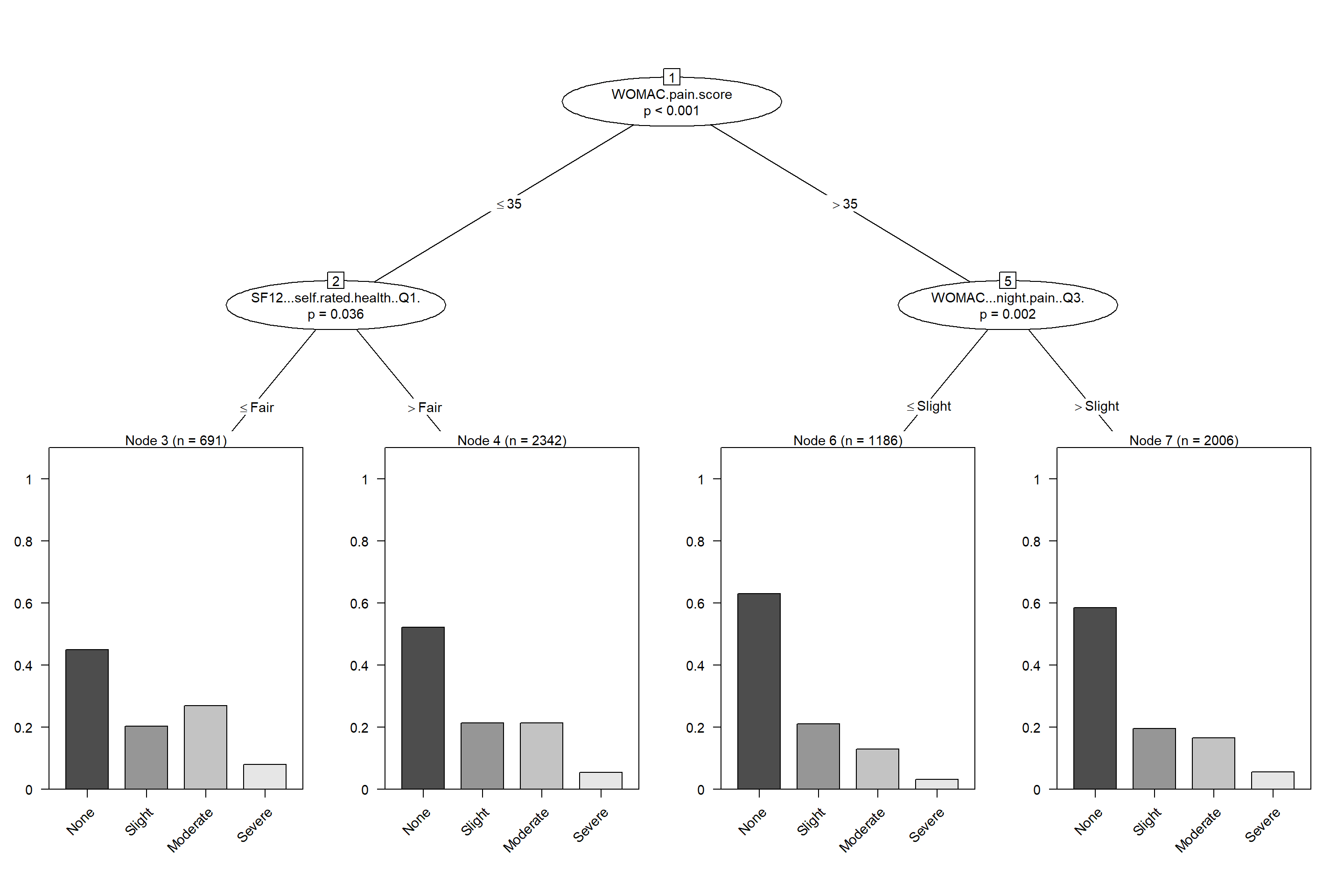


Figure 10: Night pain – year 5


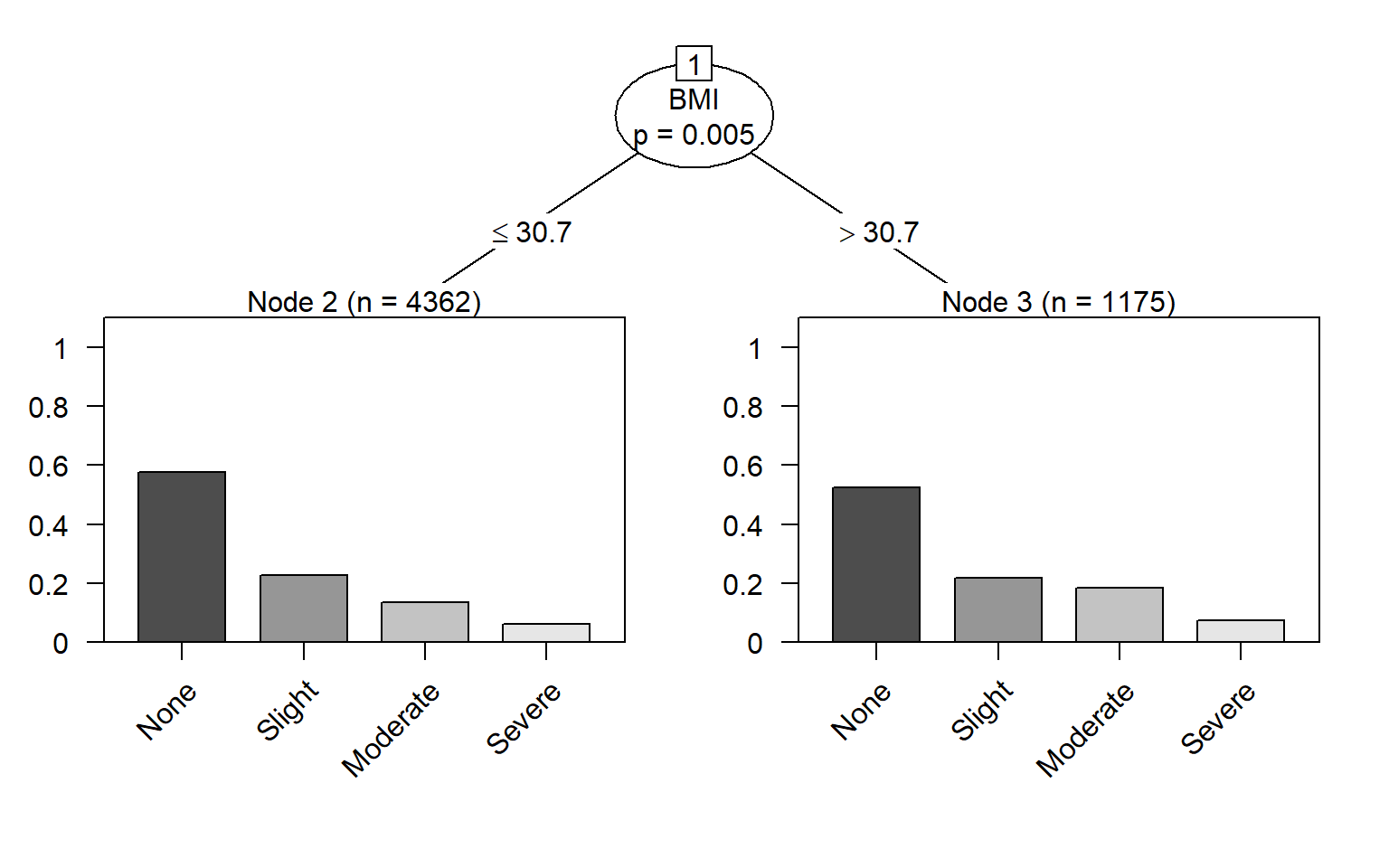


Figure 11: Night pain – year 10


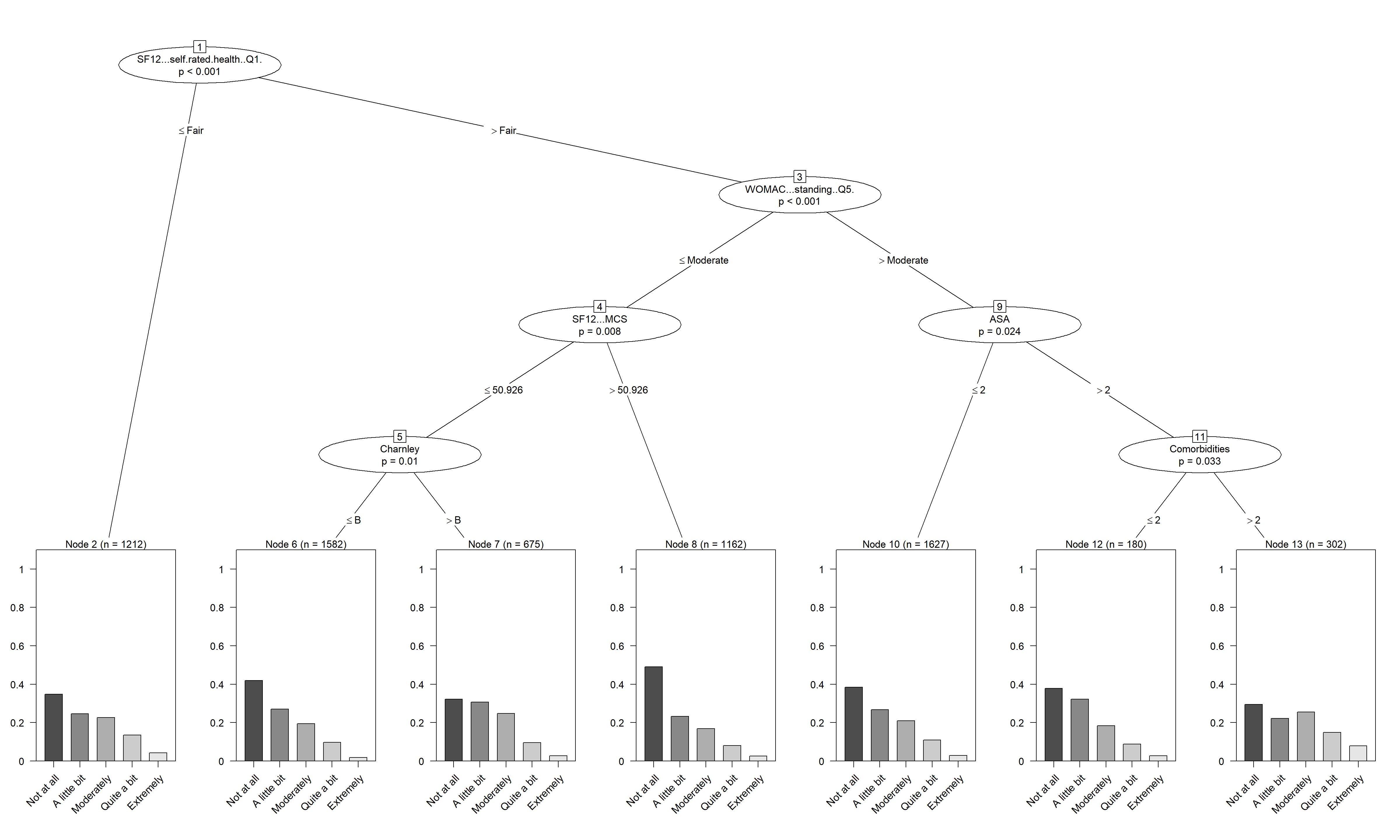


Figure 12: Pain interference – year 1


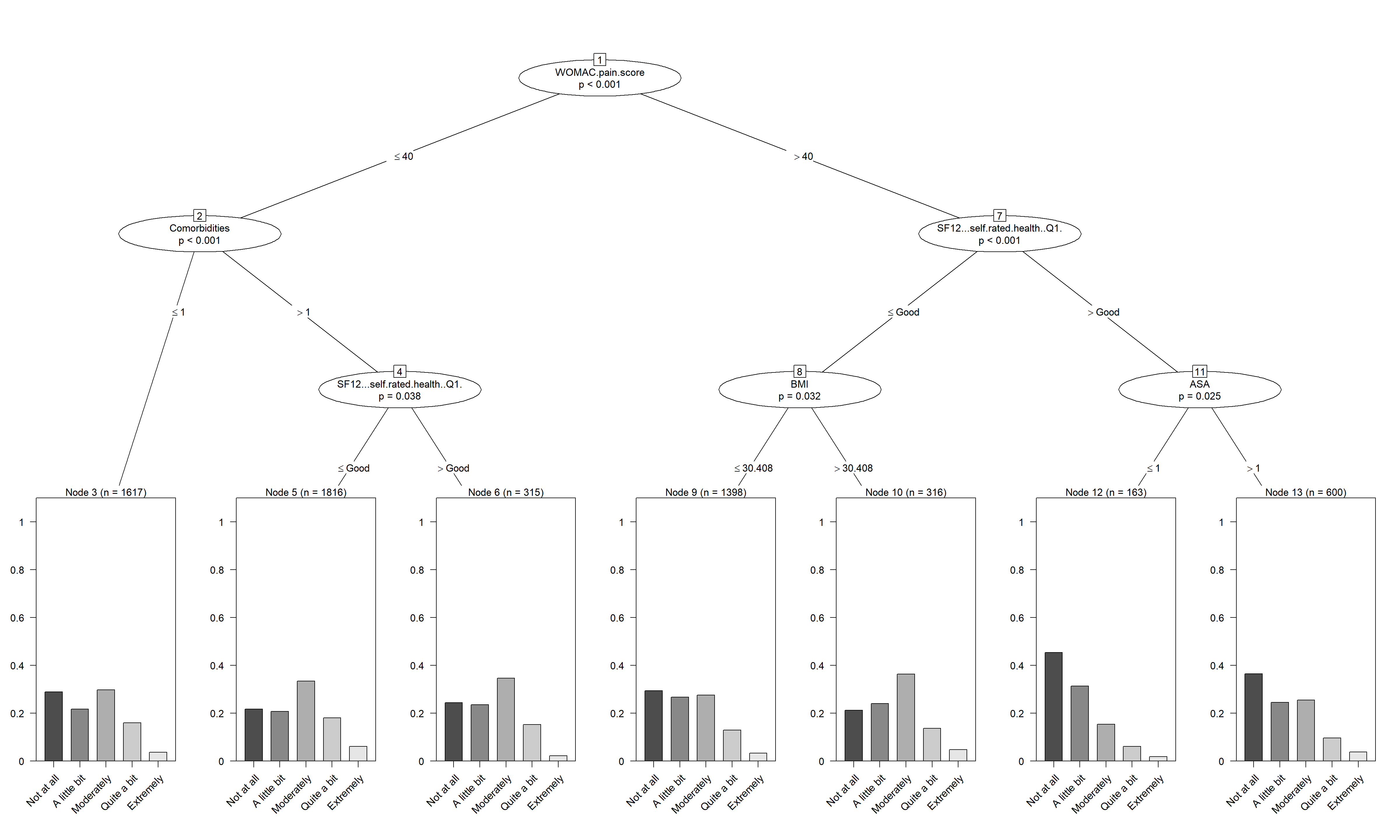


Figure 13: Pain interference – year 5


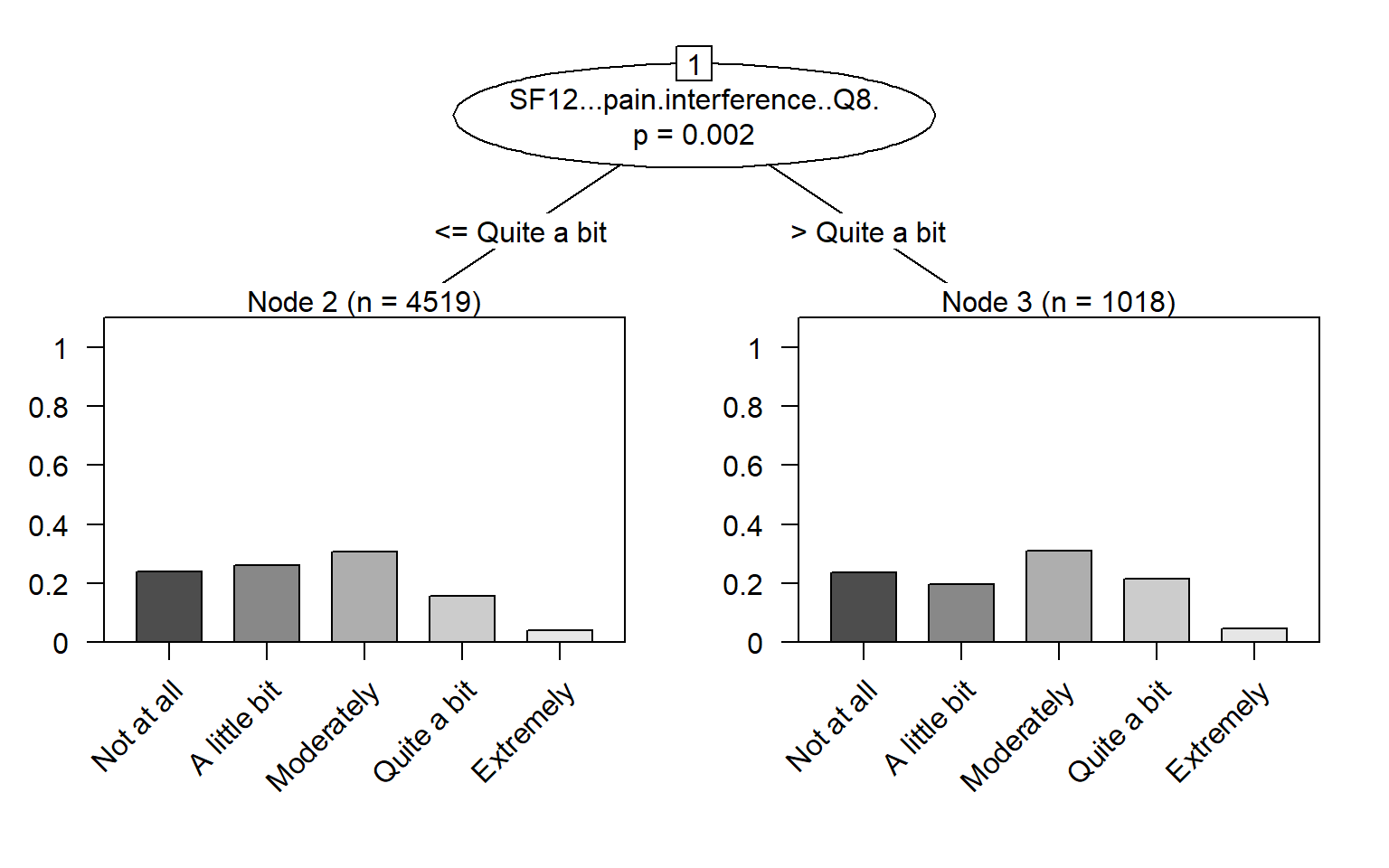


Figure 14: Pain interference – year 10


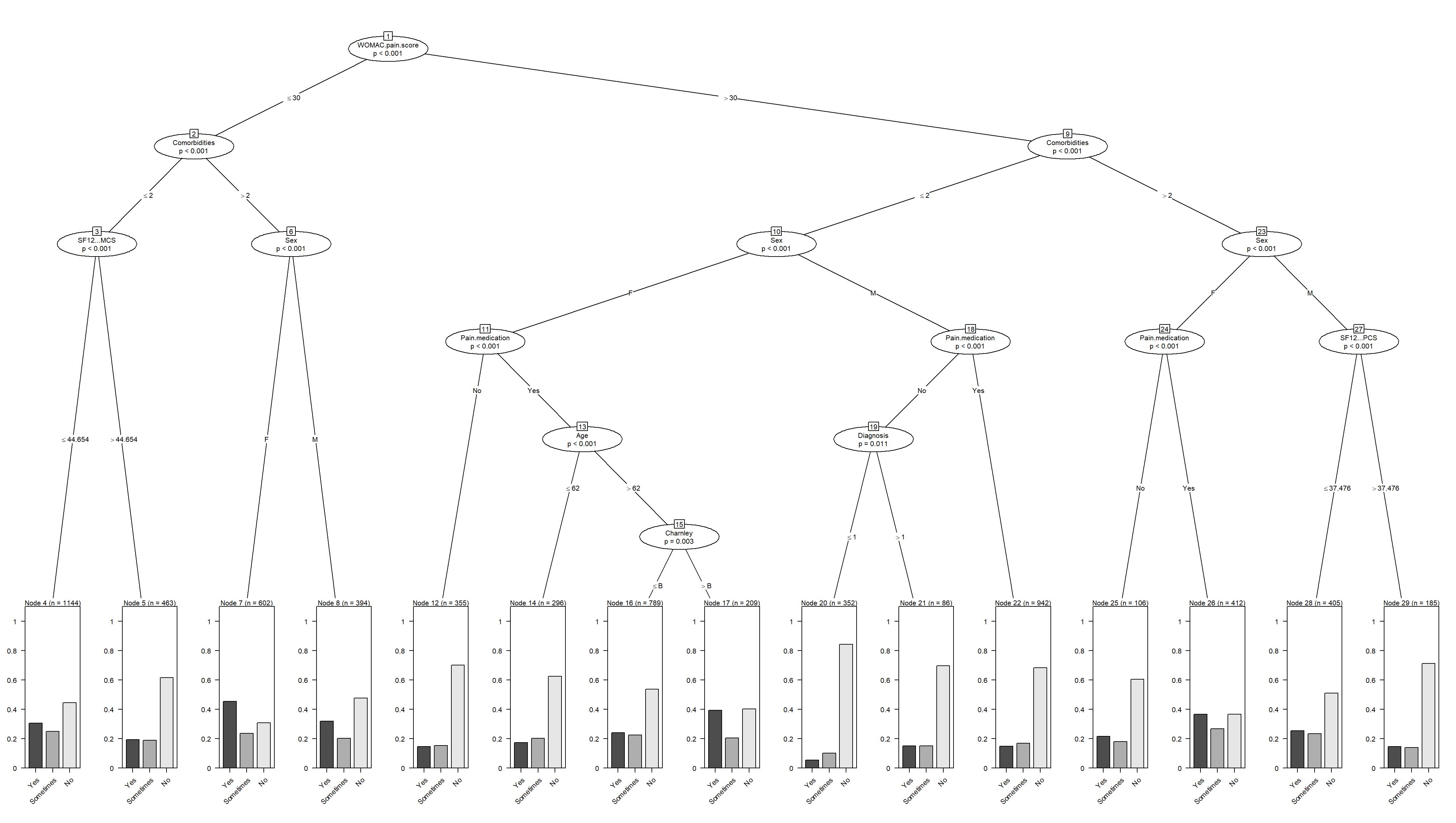


Figure 15: Pain medication – year 1


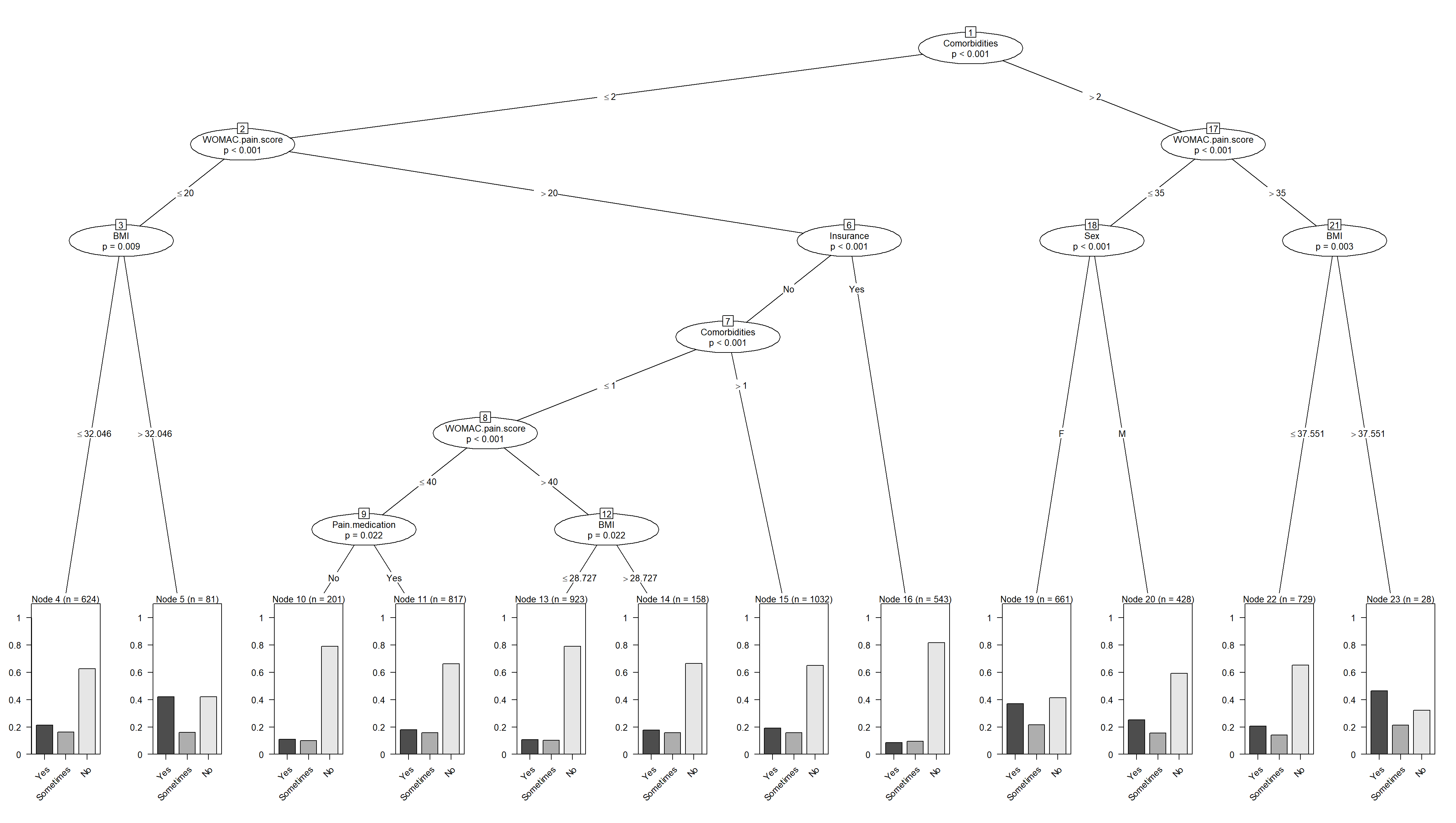


Figure 16: Pain medication – year 5


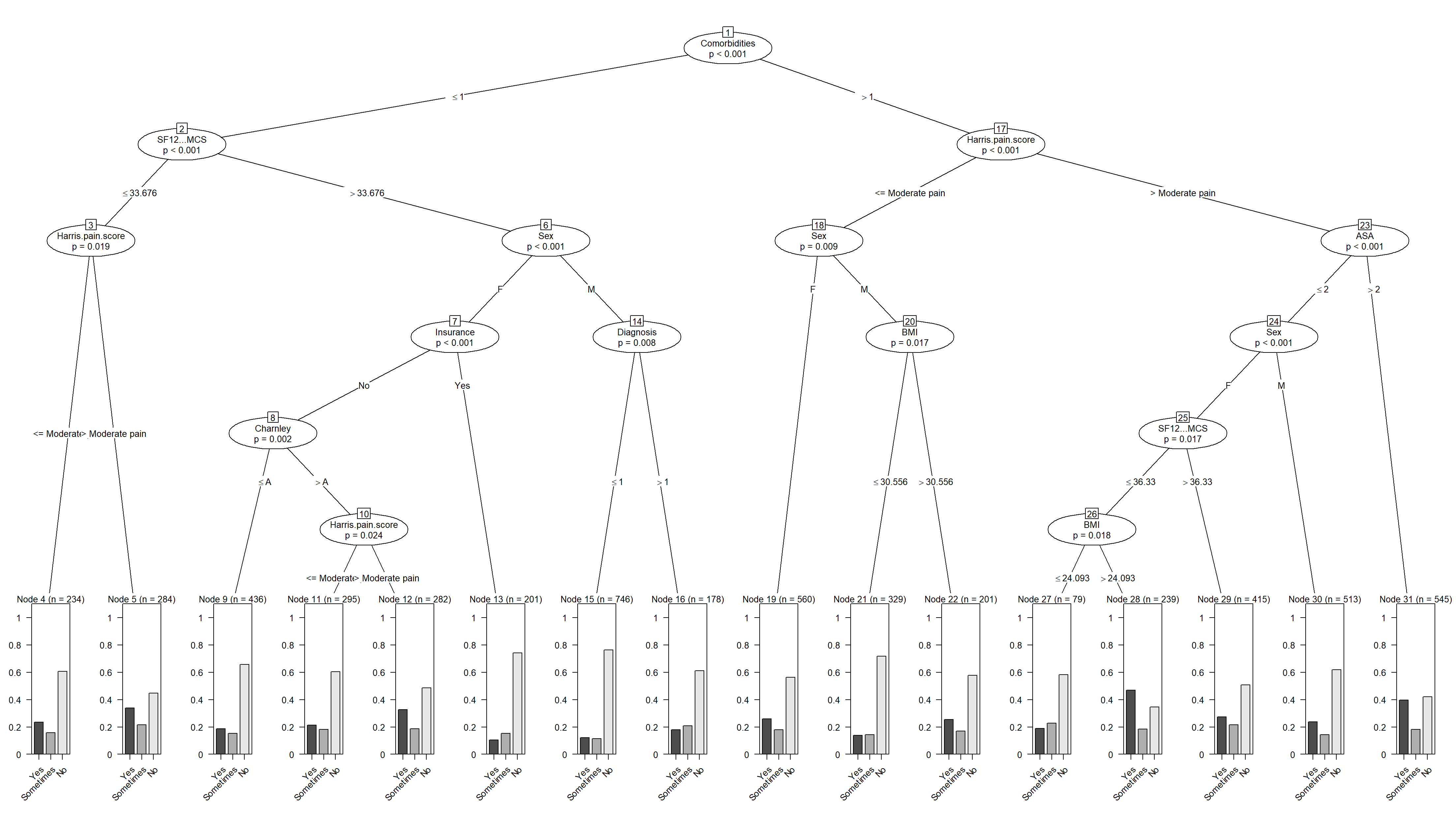


Figure 17: Pain medication – year 10

| **Age Group** | **WOMAC_Q1 Baseline** | **Observed n** | **Observed Percentage** | **Imputed n** | **Imputed Percentage** |
| --- | --- | --- | --- | --- | --- |
| <60 | None | 8 | 1.1 | 17 | 2.63 |
| <60 | Slight | 57 | 7.83 | 53 | 8.2 |
| <60 | Moderate | 284 | 39.01 | 228 | 35.29 |
| <60 | Severe | 302 | 41.48 | 263 | 40.71 |
| <60 | Extremely severe | 77 | 10.58 | 85 | 13.16 |
| 60-75 | None | 30 | 1.6 | 27 | 1.95 |
| 60-75 | Slight | 172 | 9.19 | 115 | 8.32 |
| 60-75 | Moderate | 714 | 38.16 | 532 | 38.49 |
| 60-75 | Severe | 752 | 40.19 | 552 | 39.94 |
| 60-75 | Extremely severe | 203 | 10.85 | 156 | 11.29 |
| >75 | None | 22 | 1.79 | 26 | 2.66 |
| >75 | Slight | 85 | 6.91 | 70 | 7.15 |
| >75 | Moderate | 427 | 34.72 | 366 | 37.39 |
| >75 | Severe | 530 | 43.09 | 397 | 40.55 |
| >75 | Extremely severe | 166 | 13.5 | 120 | 12.26 |

Table 3: Comparison between observed and imputed data stratified by age, WOMAC question 1, Baseline

Table 4: Comparison between observed and imputed data stratified by age, WOMAC question 1, Year 1

| **Age Group** | **WOMAC_Q1 Year 1** | **Observed n** | **Observed Percentage** | **Imputed n** | **Imputed Percentage** |
| --- | --- | --- | --- | --- | --- |
| <60 | None | 224 | 54.63 | 612 | 63.75 |
| <60 | Slight | 104 | 25.37 | 209 | 21.77 |
| <60 | Moderate | 58 | 14.15 | 94 | 9.79 |
| <60 | Severe | 20 | 4.88 | 40 | 4.17 |
| <60 | Extremely severe | 4 | 0.98 | 5 | 0.52 |
| 60-75 | None | 617 | 70.11 | 1526 | 65.13 |
| 60-75 | Slight | 153 | 17.39 | 437 | 18.65 |
| 60-75 | Moderate | 88 | 10 | 275 | 11.74 |
| 60-75 | Severe | 21 | 2.39 | 84 | 3.59 |
| 60-75 | Extremely severe | 1 | 0.11 | 21 | 0.9 |
| >75 | None | 453 | 68.64 | 980 | 65.9 |
| >75 | Slight | 97 | 14.7 | 241 | 16.21 |
| >75 | Moderate | 87 | 13.18 | 207 | 13.92 |
| >75 | Severe | 21 | 3.18 | 46 | 3.09 |
| >75 | Extremely severe | 2 | 0.3 | 13 | 0.87 |

Table 5: Comparison between observed and imputed data stratified by age, WOMAC question 1, Year 5

| **Age Group** | **WOMAC_Q1 Year 5** | **Observed n** | **Observed Percentage** | **Imputed n** | **Imputed Percentage** |
| --- | --- | --- | --- | --- | --- |
| <60 | None | 254 | 46.61 | 360 | 45.4 |
| <60 | Slight | 108 | 19.82 | 152 | 19.17 |
| <60 | Moderate | 138 | 25.32 | 230 | 29 |
| <60 | Severe | 36 | 6.61 | 42 | 5.3 |
| <60 | Extremely severe | 9 | 1.65 | 9 | 1.13 |
| 60-75 | None | 739 | 47.37 | 692 | 46.41 |
| 60-75 | Slight | 341 | 21.86 | 293 | 19.65 |
| 60-75 | Moderate | 343 | 21.99 | 362 | 24.28 |
| 60-75 | Severe | 122 | 7.82 | 114 | 7.65 |
| 60-75 | Extremely severe | 15 | 0.96 | 30 | 2.01 |
| >75 | None | 346 | 45.11 | 476 | 44.53 |
| >75 | Slight | 159 | 20.73 | 237 | 22.17 |
| >75 | Moderate | 192 | 25.03 | 238 | 22.26 |
| >75 | Severe | 54 | 7.04 | 90 | 8.42 |
| >75 | Extremely severe | 16 | 2.09 | 28 | 2.62 |

Table 6: Comparison between observed and imputed data stratified by age, WOMAC question 1, Year 10

| **Age Group** | **WOMAC_Q1 Year 10** | **Observed n** | **Observed Percentage** | **Imputed n** | **Imputed Percentage** |
| --- | --- | --- | --- | --- | --- |
| <60 | None | 132 | 46.32 | 475 | 46.39 |
| <60 | Slight | 57 | 20 | 192 | 18.75 |
| <60 | Moderate | 74 | 25.96 | 273 | 26.66 |
| <60 | Severe | 19 | 6.67 | 67 | 6.54 |
| <60 | Extremely severe | 3 | 1.05 | 17 | 1.66 |
| 60-75 | None | 378 | 46.15 | 952 | 47.7 |
| 60-75 | Slight | 175 | 21.37 | 388 | 19.44 |
| 60-75 | Moderate | 195 | 23.81 | 469 | 23.5 |
| 60-75 | Severe | 64 | 7.81 | 151 | 7.57 |
| 60-75 | Extremely severe | 7 | 0.85 | 36 | 1.8 |
| >75 | None | 112 | 53.59 | 559 | 46.43 |
| >75 | Slight | 37 | 17.7 | 244 | 20.27 |
| >75 | Moderate | 44 | 21.05 | 281 | 23.34 |
| >75 | Severe | 14 | 6.7 | 93 | 7.72 |
| >75 | Extremely severe | 2 | 0.96 | 27 | 2.24 |

Table 7: Comparison between observed and imputed data stratified by sex, WOMAC question 1, Baseline

| **Sex** | **WOMAC_Q1 Baseline** | **Observed n** | **Observed Percentage** | **Imputed n** | **Imputed Percentage** |
| --- | --- | --- | --- | --- | --- |
| F | None | 36 | 1.68 | 42 | 2.42 |
| F | Slight | 166 | 7.73 | 139 | 8 |
| F | Moderate | 755 | 35.15 | 657 | 37.8 |
| F | Severe | 900 | 41.9 | 665 | 38.26 |
| F | Extremely severe | 291 | 13.55 | 235 | 13.52 |
| M | None | 24 | 1.43 | 28 | 2.21 |
| M | Slight | 148 | 8.8 | 99 | 7.8 |
| M | Moderate | 670 | 39.86 | 469 | 36.96 |
| M | Severe | 684 | 40.69 | 547 | 43.1 |
| M | Extremely severe | 155 | 9.22 | 126 | 9.93 |

Table 8: Comparison between observed and imputed data stratified by sex, WOMAC question 1, Year 1

| **Sex** | **WOMAC_Q1 Year 1** | **Observed n** | **Observed Percentage** | **Imputed n** | **Imputed Percentage** |
| --- | --- | --- | --- | --- | --- |
| F | None | 737 | 68.43 | 1803 | 65.37 |
| F | Slight | 178 | 16.53 | 506 | 18.35 |
| F | Moderate | 120 | 11.14 | 326 | 11.82 |
| F | Severe | 37 | 3.44 | 98 | 3.55 |
| F | Extremely severe | 5 | 0.46 | 25 | 0.91 |
| M | None | 557 | 63.8 | 1315 | 64.71 |
| M | Slight | 176 | 20.16 | 381 | 18.75 |
| M | Moderate | 113 | 12.94 | 250 | 12.3 |
| M | Severe | 25 | 2.86 | 72 | 3.54 |
| M | Extremely severe | 2 | 0.23 | 14 | 0.69 |

Table 9: Comparison between observed and imputed data stratified by sex, WOMAC question 1, Year 5

| **Sex** | **WOMAC_Q1 Year 5** | **Observed n** | **Observed Percentage** | **Imputed n** | **Imputed Percentage** |
| --- | --- | --- | --- | --- | --- |
| F | None | 743 | 45.5 | 871 | 45.04 |
| F | Slight | 326 | 19.96 | 386 | 19.96 |
| F | Moderate | 404 | 24.74 | 489 | 25.28 |
| F | Severe | 135 | 8.27 | 147 | 7.6 |
| F | Extremely severe | 25 | 1.53 | 41 | 2.12 |
| M | None | 596 | 48.1 | 657 | 46.3 |
| M | Slight | 282 | 22.76 | 296 | 20.86 |
| M | Moderate | 269 | 21.71 | 341 | 24.03 |
| M | Severe | 77 | 6.21 | 99 | 6.98 |
| M | Extremely severe | 15 | 1.21 | 26 | 1.83 |

Table 10: Comparison between observed and imputed data stratified by sex, WOMAC question 1, Year 10

| **Sex** | **WOMAC_Q1 Year 10** | **Observed n** | **Observed Percentage** | **Imputed n** | **Imputed Percentage** |
| --- | --- | --- | --- | --- | --- |
| F | None | 348 | 46.28 | 1149 | 47.56 |
| F | Slight | 163 | 21.68 | 481 | 19.91 |
| F | Moderate | 178 | 23.67 | 555 | 22.97 |
| F | Severe | 56 | 7.45 | 186 | 7.7 |
| F | Extremely severe | 7 | 0.93 | 45 | 1.86 |
| M | None | 274 | 48.84 | 837 | 46.29 |
| M | Slight | 106 | 18.89 | 343 | 18.97 |
| M | Moderate | 135 | 24.06 | 468 | 25.88 |
| M | Severe | 41 | 7.31 | 125 | 6.91 |
| M | Extremely severe | 5 | 0.89 | 35 | 1.94 |

Table 11: Comparison between observed and imputed data stratified by BMI, WOMAC question 1, Baseline

| **BMI group** | **WOMAC_Q1 Baseline** | **Observed n** | **Observed Percentage** | **Imputed n** | **Imputed Percentage** |
| --- | --- | --- | --- | --- | --- |
| Healthy weight | None | 25 | 1.89 | 28 | 2.54 |
| Healthy weight | Slight | 132 | 9.95 | 94 | 8.53 |
| Healthy weight | Moderate | 510 | 38.46 | 404 | 36.66 |
| Healthy weight | Severe | 527 | 39.74 | 443 | 40.2 |
| Healthy weight | Extremely severe | 132 | 9.95 | 133 | 12.07 |
| Obese | None | 7 | 0.77 | 20 | 2.83 |
| Obese | Slight | 55 | 6.05 | 57 | 8.06 |
| Obese | Moderate | 294 | 32.34 | 265 | 37.48 |
| Obese | Severe | 418 | 45.98 | 272 | 38.47 |
| Obese | Extremely severe | 135 | 14.85 | 93 | 13.15 |
| Overweight | None | 22 | 1.5 | 20 | 1.83 |
| Overweight | Slight | 118 | 8.02 | 79 | 7.25 |
| Overweight | Moderate | 584 | 39.7 | 415 | 38.07 |
| Overweight | Severe | 588 | 39.97 | 453 | 41.56 |
| Overweight | Extremely severe | 159 | 10.81 | 123 | 11.28 |
| Severely obese | None | 3 | 5.56 |  |  |
| Severely obese | Slight | 2 | 3.7 | 4 | 10.53 |
| Severely obese | Moderate | 14 | 25.93 | 16 | 42.11 |
| Severely obese | Severe | 26 | 48.15 | 13 | 34.21 |
| Severely obese | Extremely severe | 9 | 16.67 | 5 | 13.16 |
| Underweight | None | 3 | 4.35 | 2 | 2.86 |
| Underweight | Slight | 7 | 10.14 | 4 | 5.71 |
| Underweight | Moderate | 23 | 33.33 | 26 | 37.14 |
| Underweight | Severe | 25 | 36.23 | 31 | 44.29 |
| Underweight | Extremely severe | 11 | 15.94 | 7 | 10 |

Table 12: Comparison between observed and imputed data stratified by BMI, WOMAC question 1, Year 1

| **BMI group** | **WOMAC_Q1 Year 1** | **Observed n** | **Observed Percentage** | **Imputed n** | **Imputed Percentage** |
| --- | --- | --- | --- | --- | --- |
| Healthy weight | None | 484 | 69.24 | 1117 | 66.02 |
| Healthy weight | Slight | 114 | 16.31 | 298 | 17.61 |
| Healthy weight | Moderate | 88 | 12.59 | 190 | 11.23 |
| Healthy weight | Severe | 9 | 1.29 | 70 | 4.14 |
| Healthy weight | Extremely severe | 4 | 0.57 | 17 | 1 |
| Obese | None | 267 | 60.27 | 761 | 65.77 |
| Obese | Slight | 87 | 19.64 | 219 | 18.93 |
| Obese | Moderate | 66 | 14.9 | 137 | 11.84 |
| Obese | Severe | 22 | 4.97 | 34 | 2.94 |
| Obese | Extremely severe | 1 | 0.23 | 6 | 0.52 |
| Overweight | None | 486 | 66.76 | 1141 | 63.6 |
| Overweight | Slight | 138 | 18.96 | 345 | 19.23 |
| Overweight | Moderate | 74 | 10.16 | 231 | 12.88 |
| Overweight | Severe | 29 | 3.98 | 62 | 3.46 |
| Overweight | Extremely severe | 1 | 0.14 | 15 | 0.84 |
| Severely obese | None | 20 | 58.82 | 36 | 64.29 |
| Severely obese | Slight | 8 | 23.53 | 10 | 17.86 |
| Severely obese | Moderate | 4 | 11.76 | 8 | 14.29 |
| Severely obese | Severe | 1 | 2.94 | 1 | 1.79 |
| Severely obese | Extremely severe | 1 | 2.94 | 1 | 1.79 |
| Underweight | None | 37 | 80.43 | 63 | 69.23 |
| Underweight | Slight | 7 | 15.22 | 15 | 16.48 |
| Underweight | Moderate | 1 | 2.17 | 10 | 10.99 |
| Underweight | Severe | 1 | 2.17 | 3 | 3.3 |

Table 13: Comparison between observed and imputed data stratified by BMI, WOMAC question 1, Year 5

| **BMI group** | **WOMAC_Q1 Year 5** | **Observed n** | **Observed Percentage** | **Imputed n** | **Imputed Percentage** |
| --- | --- | --- | --- | --- | --- |
| Healthy weight | None | 521 | 52.36 | 556 | 46.22 |
| Healthy weight | Slight | 201 | 20.2 | 255 | 21.2 |
| Healthy weight | Moderate | 217 | 21.81 | 288 | 23.94 |
| Healthy weight | Severe | 46 | 4.62 | 89 | 7.4 |
| Healthy weight | Extremely severe | 10 | 1.01 | 15 | 1.25 |
| Obese | None | 267 | 40.45 | 367 | 44.43 |
| Obese | Slight | 134 | 20.3 | 165 | 19.98 |
| Obese | Moderate | 167 | 25.3 | 205 | 24.82 |
| Obese | Severe | 72 | 10.91 | 65 | 7.87 |
| Obese | Extremely severe | 20 | 3.03 | 24 | 2.91 |
| Overweight | None | 518 | 45.24 | 538 | 45.02 |
| Overweight | Slight | 260 | 22.71 | 242 | 20.25 |
| Overweight | Moderate | 270 | 23.58 | 305 | 25.52 |
| Overweight | Severe | 88 | 7.69 | 86 | 7.2 |
| Overweight | Extremely severe | 9 | 0.79 | 24 | 2.01 |
| Severely obese | None | 6 | 24 | 34 | 55.74 |
| Severely obese | Slight | 4 | 16 | 4 | 6.56 |
| Severely obese | Moderate | 9 | 36 | 17 | 27.87 |
| Severely obese | Severe | 5 | 20 | 3 | 4.92 |
| Severely obese | Extremely severe | 1 | 4 | 3 | 4.92 |
| Underweight | None | 27 | 57.45 | 33 | 48.53 |
| Underweight | Slight | 9 | 19.15 | 16 | 23.53 |
| Underweight | Moderate | 10 | 21.28 | 15 | 22.06 |
| Underweight | Severe | 1 | 2.13 | 3 | 4.41 |
| Underweight | Extremely severe |  |  | 1 | 1.47 |

Table 14: Comparison between observed and imputed data stratified by BMI, WOMAC question 1, Year 10

| **BMI group** | **WOMAC_Q1 Year 10** | **Observed n** | **Observed Percentage** | **Imputed n** | **Imputed Percentage** |
| --- | --- | --- | --- | --- | --- |
| Healthy weight | None | 220 | 51.89 | 698 | 46.29 |
| Healthy weight | Slight | 87 | 20.52 | 295 | 19.56 |
| Healthy weight | Moderate | 97 | 22.88 | 360 | 23.87 |
| Healthy weight | Severe | 19 | 4.48 | 123 | 8.16 |
| Healthy weight | Extremely severe | 1 | 0.24 | 32 | 2.12 |
| Obese | None | 128 | 39.88 | 460 | 45.59 |
| Obese | Slight | 68 | 21.18 | 196 | 19.43 |
| Obese | Moderate | 90 | 28.04 | 254 | 25.17 |
| Obese | Severe | 32 | 9.97 | 75 | 7.43 |
| Obese | Extremely severe | 3 | 0.93 | 24 | 2.38 |
| Overweight | None | 264 | 48.62 | 753 | 48.33 |
| Overweight | Slight | 107 | 19.71 | 306 | 19.64 |
| Overweight | Moderate | 123 | 22.65 | 371 | 23.81 |
| Overweight | Severe | 42 | 7.73 | 106 | 6.8 |
| Overweight | Extremely severe | 7 | 1.29 | 22 | 1.41 |
| Severely obese | None | 6 | 60 | 37 | 53.62 |
| Severely obese | Slight | 1 | 10 | 11 | 15.94 |
| Severely obese | Severe | 2 | 20 | 4 | 5.8 |
| Severely obese | Extremely severe | 1 | 10 |  |  |
| Severely obese | Moderate |  |  | 17 | 24.64 |
| Underweight | None | 4 | 26.67 | 38 | 47.5 |
| Underweight | Slight | 6 | 40 | 16 | 20 |
| Underweight | Moderate | 3 | 20 | 21 | 26.25 |
| Underweight | Severe | 2 | 13.33 | 3 | 3.75 |
| Underweight | Extremely severe |  |  | 2 | 2.5 |

Table 15: Comparison between observed and imputed data stratified by age, Pain medication, Baseline

| **Age** | **Pain medication Baseline** | **Observed n** | **Observed Percentage** | **Imputed n** | **Imputed Percentage** |
| --- | --- | --- | --- | --- | --- |
| <60 | No | 74 | 21.33 | 225 | 21.91 |
| <60 | Yes | 273 | 78.67 | 802 | 78.09 |
| 60-75 | No | 132 | 17.81 | 548 | 21.82 |
| 60-75 | Yes | 609 | 82.19 | 1964 | 78.18 |
| >75 | No | 78 | 14.53 | 312 | 18.66 |
| >75 | Yes | 459 | 85.47 | 1360 | 81.34 |

Table 16: Comparison between observed and imputed data stratified by age, Pain medication, Year 1

| **Age** | **Pain medication Year 1** | **Observed n** | **Observed Percentage** | **Imputed n** | **Imputed Percentage** |
| --- | --- | --- | --- | --- | --- |
| <60 | Yes | 89 | 22.76 | 188 | 19.2 |
| <60 | Sometimes | 81 | 20.72 | 198 | 20.22 |
| <60 | No | 221 | 56.52 | 587 | 59.96 |
| <60 | Yes/Sometimes |  |  | 3 | 0.31 |
| <60 | Sometimes/No |  |  | 3 | 0.31 |
| 60-75 | Yes | 176 | 21.39 | 573 | 23.88 |
| 60-75 | Yes/Sometimes | 3 | 0.36 | 10 | 0.42 |
| 60-75 | Sometimes | 161 | 19.56 | 494 | 20.58 |
| 60-75 | Sometimes/No | 1 | 0.12 | 12 | 0.5 |
| 60-75 | No | 482 | 58.57 | 1311 | 54.62 |
| >75 | Yes | 207 | 32.75 | 455 | 30.03 |
| >75 | Sometimes | 125 | 19.78 | 301 | 19.87 |
| >75 | No | 300 | 47.47 | 737 | 48.65 |
| >75 | Yes/Sometimes |  |  | 12 | 0.79 |
| >75 | Sometimes/No |  |  | 10 | 0.66 |

Table 17: Comparison between observed and imputed data stratified by age, Pain medication, Year 5

| **Age** | **Pain medication Year 5** | **Observed n** | **Observed Percentage** | **Imputed n** | **Imputed Percentage** |
| --- | --- | --- | --- | --- | --- |
| <60 | Yes | 104 | 18.37 | 140 | 18.13 |
| <60 | Sometimes | 88 | 15.55 | 110 | 14.25 |
| <60 | No | 374 | 66.08 | 522 | 67.62 |
| 60-75 | Yes | 269 | 17.6 | 311 | 20.42 |
| 60-75 | Sometimes | 224 | 14.66 | 220 | 14.45 |
| 60-75 | No | 1035 | 67.74 | 992 | 65.13 |
| >75 | Yes | 161 | 23.1 | 237 | 20.81 |
| >75 | Sometimes | 87 | 12.48 | 189 | 16.59 |
| >75 | No | 449 | 64.42 | 713 | 62.6 |

Table 18: Comparison between observed and imputed data stratified by age, Pain medication, Year 10

| **Age** | **Pain medication Year 10** | **Observed n** | **Observed Percentage** | **Imputed n** | **Imputed Percentage** |
| --- | --- | --- | --- | --- | --- |
| <60 | Yes | 58 | 21.48 | 220 | 21.17 |
| <60 | Sometimes | 48 | 17.78 | 165 | 15.88 |
| <60 | No | 164 | 60.74 | 654 | 62.95 |
| 60-75 | Yes | 150 | 20.13 | 532 | 25.7 |
| 60-75 | Sometimes | 117 | 15.7 | 347 | 16.76 |
| 60-75 | No | 478 | 64.16 | 1191 | 57.54 |
| >75 | Yes | 51 | 28.49 | 341 | 27.63 |
| >75 | Sometimes | 33 | 18.44 | 222 | 17.99 |
| >75 | No | 95 | 53.07 | 671 | 54.38 |

Table 19: Comparison between observed and imputed data stratified by sex, Pain medication, Baseline

| **Sex** | **Pain medication Baseline** | **Observed n** | **Observed Percentage** | **Imputed n** | **Imputed Percentage** |
| --- | --- | --- | --- | --- | --- |
| F | No | 124 | 13.61 | 510 | 17.14 |
| F | Yes | 787 | 86.39 | 2465 | 82.86 |
| M | No | 160 | 22.41 | 575 | 25.72 |
| M | Yes | 554 | 77.59 | 1661 | 74.28 |

Table 20: Comparison between observed and imputed data stratified by sex, Pain medication, Year 1

| **Sex** | **Pain medication Year 1** | **Observed n** | **Observed Percentage** | **Imputed n** | **Imputed Percentage** |
| --- | --- | --- | --- | --- | --- |
| F | Yes | 309 | 29.86 | 827 | 29.54 |
| F | Yes/Sometimes | 3 | 0.29 | 20 | 0.71 |
| F | Sometimes | 228 | 22.03 | 617 | 22.04 |
| F | No | 495 | 47.83 | 1318 | 47.07 |
| F | Sometimes/No |  |  | 18 | 0.64 |
| M | Yes | 163 | 20.1 | 389 | 18.58 |
| M | Sometimes | 139 | 17.14 | 376 | 17.96 |
| M | Sometimes/No | 1 | 0.12 | 7 | 0.33 |
| M | No | 508 | 62.64 | 1317 | 62.89 |
| M | Yes/Sometimes |  |  | 5 | 0.24 |

Table 21: Comparison between observed and imputed data stratified by sex, Pain medication, Year 5

| **Sex** | **Pain medication Year 5** | **Observed n** | **Observed Percentage** | **Imputed n** | **Imputed Percentage** |
| --- | --- | --- | --- | --- | --- |
| F | Yes | 333 | 21.1 | 434 | 21.82 |
| F | Sometimes | 252 | 15.97 | 329 | 16.54 |
| F | No | 993 | 62.93 | 1226 | 61.64 |
| M | Yes | 201 | 16.57 | 254 | 17.58 |
| M | Sometimes | 147 | 12.12 | 190 | 13.15 |
| M | No | 865 | 71.31 | 1001 | 69.27 |

Table 22: Comparison between observed and imputed data stratified by sex, Pain medication, Year 10

| **Sex** | **Pain medication Year 10** | **Observed n** | **Observed Percentage** | **Imputed n** | **Imputed Percentage** |
| --- | --- | --- | --- | --- | --- |
| F | Yes | 167 | 24.96 | 716 | 28.65 |
| F | Sometimes | 113 | 16.89 | 474 | 18.97 |
| F | No | 389 | 58.15 | 1309 | 52.38 |
| M | Yes | 92 | 17.52 | 377 | 20.44 |
| M | Sometimes | 85 | 16.19 | 260 | 14.1 |
| M | No | 348 | 66.29 | 1207 | 65.46 |

Table 23: Comparison between observed and imputed data stratified by BMI, Pain medication, Baseline

| **BMI group** | **Pain medication Baseline** | **Observed n** | **Observed Percentage** | **Imputed n** | **Imputed Percentage** |
| --- | --- | --- | --- | --- | --- |
| Healthy weight | No | 105 | 18.58 | 385 | 20.67 |
| Healthy weight | Yes | 460 | 81.42 | 1478 | 79.33 |
| Obese | No | 59 | 15.49 | 230 | 18.62 |
| Obese | Yes | 322 | 84.51 | 1005 | 81.38 |
| Overweight | No | 109 | 17.75 | 440 | 22.6 |
| Overweight | Yes | 505 | 82.25 | 1507 | 77.4 |
| Severely obese | No | 4 | 13.33 | 10 | 16.13 |
| Severely obese | Yes | 26 | 86.67 | 52 | 83.87 |
| Underweight | No | 7 | 20 | 20 | 19.23 |
| Underweight | Yes | 28 | 80 | 84 | 80.77 |

Table 24: Comparison between observed and imputed data stratified by BMI, Pain medication, Year 1

| **BMI group** | **Pain medication Year 1** | **Observed n** | **Observed Percentage** | **Imputed n** | **Imputed Percentage** |
| --- | --- | --- | --- | --- | --- |
| Healthy weight | Yes | 144 | 21.95 | 379 | 21.84 |
| Healthy weight | Yes/Sometimes | 2 | 0.3 | 7 | 0.4 |
| Healthy weight | Sometimes | 136 | 20.73 | 344 | 19.83 |
| Healthy weight | No | 374 | 57.01 | 996 | 57.41 |
| Healthy weight | Sometimes/No |  |  | 9 | 0.52 |
| Obese | Yes | 128 | 30.77 | 346 | 29.22 |
| Obese | Sometimes | 94 | 22.6 | 259 | 21.88 |
| Obese | No | 194 | 46.63 | 562 | 47.47 |
| Obese | Yes/Sometimes |  |  | 9 | 0.76 |
| Obese | Sometimes/No |  |  | 8 | 0.68 |
| Overweight | Yes | 178 | 25.46 | 450 | 24.68 |
| Overweight | Yes/Sometimes | 1 | 0.14 | 9 | 0.49 |
| Overweight | Sometimes | 125 | 17.88 | 347 | 19.03 |
| Overweight | Sometimes/No | 1 | 0.14 | 7 | 0.38 |
| Overweight | No | 394 | 56.37 | 1010 | 55.4 |
| Severely obese | Yes | 12 | 37.5 | 18 | 31.03 |
| Severely obese | Sometimes | 7 | 21.88 | 15 | 25.86 |
| Severely obese | No | 13 | 40.62 | 25 | 43.1 |
| Underweight | Yes | 10 | 23.26 | 23 | 24.47 |
| Underweight | Sometimes | 5 | 11.63 | 28 | 29.79 |
| Underweight | No | 28 | 65.12 | 42 | 44.68 |
| Underweight | Sometimes/No |  |  | 1 | 1.06 |

Table 25: Comparison between observed and imputed data stratified by BMI, Pain medication, Year 5

| **BMI group** | **Pain medication Year 5** | **Observed n** | **Observed Percentage** | **Imputed n** | **Imputed Percentage** |
| --- | --- | --- | --- | --- | --- |
| Healthy weight | Yes | 152 | 15.75 | 198 | 16.06 |
| Healthy weight | Sometimes | 109 | 11.3 | 187 | 15.17 |
| Healthy weight | No | 704 | 72.95 | 848 | 68.78 |
| Obese | Yes | 160 | 24.81 | 229 | 27.23 |
| Obese | Sometimes | 111 | 17.21 | 135 | 16.05 |
| Obese | No | 374 | 57.98 | 477 | 56.72 |
| Overweight | Yes | 197 | 17.86 | 233 | 18.84 |
| Overweight | Sometimes | 171 | 15.5 | 172 | 13.9 |
| Overweight | No | 735 | 66.64 | 832 | 67.26 |
| Severely obese | Yes | 17 | 53.12 | 20 | 37.04 |
| Severely obese | Sometimes | 5 | 15.62 | 13 | 24.07 |
| Severely obese | No | 10 | 31.25 | 21 | 38.89 |
| Underweight | Yes | 8 | 17.39 | 8 | 11.59 |
| Underweight | Sometimes | 3 | 6.52 | 12 | 17.39 |
| Underweight | No | 35 | 76.09 | 49 | 71.01 |

Table 26: Comparison between observed and imputed data stratified by BMI, Pain medication, Year 10

| **BMI group** | **Pain medication Year 10** | **Observed n** | **Observed Percentage** | **Imputed n** | **Imputed Percentage** |
| --- | --- | --- | --- | --- | --- |
| Healthy weight | Yes | 75 | 18.66 | 350 | 22.88 |
| Healthy weight | Sometimes | 66 | 16.42 | 254 | 16.6 |
| Healthy weight | No | 261 | 64.93 | 926 | 60.52 |
| Obese | Yes | 67 | 23.67 | 303 | 28.94 |
| Obese | Sometimes | 48 | 16.96 | 184 | 17.57 |
| Obese | No | 168 | 59.36 | 560 | 53.49 |
| Overweight | Yes | 112 | 22.9 | 397 | 24.63 |
| Overweight | Sometimes | 81 | 16.56 | 260 | 16.13 |
| Overweight | No | 296 | 60.53 | 955 | 59.24 |
| Severely obese | Yes | 3 | 50 | 29 | 39.73 |
| Severely obese | Sometimes | 1 | 16.67 | 19 | 26.03 |
| Severely obese | No | 2 | 33.33 | 25 | 34.25 |
| Underweight | Yes | 2 | 14.29 | 14 | 17.28 |
| Underweight | Sometimes | 2 | 14.29 | 17 | 20.99 |
| Underweight | No | 10 | 71.43 | 50 | 61.73 |

1. **References:**

1. Regidor, E., et al., *Socioeconomic patterns in the use of public and private health services and equity in health care.* BMC health services research, 2008. **8**(1): p. 1-9.

2. Khan, L.K., et al., *The complication rate and medium-term functional outcome after total hip replacement in smokers.* Hip International, 2009. **19**(1): p. 47-51.

3. Matharu, G.S., et al., *The effect of smoking on outcomes following primary total hip and knee arthroplasty: a population-based cohort study of 117,024 patients.* Acta orthopaedica, 2019. **90**(6): p. 559-567.

4. Lungu, E., et al., *A systematic review of preoperative determinants of patient-reported pain and physical function up to 2 years following primary unilateral total hip arthroplasty.* Orthopaedics & Traumatology: Surgery & Research, 2016. **102**(3): p. 397-403.

5. Van Buuren, S. and K. Groothuis-Oudshoorn, *mice: Multivariate imputation by chained equations in R.* Journal of statistical software, 2011. **45**: p. 1-67.
